# A systematic review and meta-analysis of protozoan parasite infections among patients with mental health disorders: an overlooked phenomenon

**DOI:** 10.1186/s13099-024-00602-2

**Published:** 2024-01-28

**Authors:** Amir Abdoli, Meysam Olfatifar, Aida Vafae Eslahi, Zeinab Moghadamizad, Rasoul Samimi, Mohammad Amin Habibi, Amir Sam Kianimoghadam, Milad Badri, Panagiotis Karanis

**Affiliations:** 1 Zoonoses Research Center, Jahrom University of Medical Sciences, Jahrom, Iran; 2 Department of Parasitology and Mycology, Jahrom University of Medical Sciences, Jahrom, Iran; 3 Gastroenterology and Hepatology Diseases Research Center, Qom University of Medical Sciences, Qom, Iran; 4 Medical Microbiology Research Center, Qazvin University of Medical Sciences, Qazvin, Iran; 5 Department of Parasitology, Faculty of Medical Sciences, Tarbiat Modares University, Tehran, Iran; 6 Skull Base Research Center, Loghman Hakim Hospital, Shahid Beheshti University of Medical Sciences, Tehran, Iran; 7 Clinical Research Development Center, Qom University of Medical Sciences, Qom, Iran; 8 Department of Clinical Psychology, School of Medicine, Shahid Beheshti University of Medical Sciences, Tehran, Iran; 9 University of Cologne, Medical Faculty and University Hospital, Cologne, Germany; 10 Medical School, Department of Basic and Clinical Sciences, Anatomy Centre, University of Nicosia, Nicosia, Cyprus

**Keywords:** Protozoan parasites, Mental disorders, Prevalence, Meta-analysis, Worldwide

## Abstract

**Background:**

Patients with mental disorders have a high risk of intestinal parasitic infection due to poor hygiene practices. Hence, to better clarify this overlooked phenomenon, the current study is conducted to determine the global prevalence of protozoan parasite infections in patients with mental disorders and investigate the associated risk factors.

**Methods:**

Several databases (PubMed, Scopus, Web of Science, ProQuest, and Google Scholar) were searched for papers published until December 2022. The fixed effect meta-analysis was used to estimate the overall odds ratio (OR) and pooled prevalence was estimated using a random-effects model with a 95% confidence interval (CI).

**Results:**

Totally, 131 articles (91 case–control and 40 cross-sectional studies) met the eligibility criteria. Patients with mental disorders were significantly at higher risk for protozoan parasites than healthy controls (OR: 2.059, 1.830–2.317). The highest pooled OR (2.485, 1.413–4.368) was related to patients with neurodevelopmental disorders, and the highest pooled prevalence was detected in patients with neurodevelopmental disorders (0.341, 0.244–0.446), followed by bipolar and related disorders (0.321, 0.000–0.995). *Toxoplasma gondii* was the most prevalent protozoan parasite (0.343, 0.228–0.467) in cross-sectional studies and the highest pooled OR was related to *Cyclospora cayetanensis* (4.719, 1.352–16.474) followed by *Cryptosporidium parvum* (4.618, 2.877–7.412).

**Conclusion:**

Our findings demonstrated that individuals afflicted with mental disorders are significantly more susceptible to acquiring protozoan parasites in comparison to healthy individuals. Preventive interventions, regular screening, and treatment approaches for parasitic diseases should be considered for patients with mental disorders.

**Supplementary Information:**

The online version contains supplementary material available at 10.1186/s13099-024-00602-2.

## Background

Over the past few decades, protozoan parasites have been recognized as significant potential agents to cause waterborne and foodborne disease [1, 2]. Enteric protozoa of public health importance are associated with diarrheal illnesses, contributing to severe morbidity and mortality in both humans and animals globally [3]. Approximately 3.5 billion people in the world are infected by enteric protozoan parasites, which are responsible for 1.7 billion annual diarrhea cases [4]. Intestinal parasitic infections are a highly prevalent and significant health concern worldwide, particularly in developing countries. The diarrhoea caused by these pathogens can be chronic or severe, with clinical symptoms including abdominal cramps, nausea, vomiting, anorexia, weight loss, fatigue, and a mild fever [5].

Mental disorders are common health issues affecting people of all ages worldwide [6, 7]. According to WHO, in 2019, 970 million people were living with a mental disorder globally [8]. Psychiatric diseases, also called mental diseases are psychological and behavioural syndromes that account for 30% of the global disease burden. They cover many mental health conditions but mainly refer to disorders influencing emotions, thinking, and behavior. Depression, anxiety and hypochondriasis disorders, dementia, schizophrenia, autism spectrum disorders, and personality disorders are examples of mental disorders [9].

Patients with mental disorders can be more susceptible to infections due to a combination of factors that influence their immune system, lifestyle, and overall health. The conditions associated with mental diseases might lead to neglect of personal hygiene, a lack of motivation to seek medical care, or limited healthcare access. Furthermore, mental disorders can affect the functions of the immune system (e.g., due to malnutrition, sleep disruption, chronic stress, medication side effects, neuroendocrine effects, and chronic inflammation), leading to dysregulation and diminished immune responses, making individuals more susceptible to infections [10, 11].

Mental disorders are considered risk factors leading to parasitic infections because the lack of hygienic behaviours in psychiatric patients. Keeping these patients in close contact with each other as the condition occurs through institutionalization can intensify the risk of acquiring a disease, primarily when the environment is confined and the sanitation level is poor [12, 13].

There are several mental symptoms with unknown aetiologies, which may be due to microbial pathogens. Many infectious diseases are often connected with severe behavioral problems, including depression, decreased physical and social activities, hyposomnia or hypersomnia, anorexia, malaise, fatigue, and cognitive disturbances [9]. Based on a review, mental illnesses are common in low and middle-income countries, with a pooled prevalence of 17.6% (15.5–20.0%) [14]. There are neurotropic parasitic diseases including malaria, toxoplasmosis, African human trypanosomiasis, Chagas disease, cysticercosis, and human toxocariasis [15, 16].

Parasitic diseases with neurological effects are regarded as public health issues widely prevalent in developing countries, where more than 25% of population encounters the development of one or multiple mental or behavioral disorders in their lifetime [17].

Despite the high number of reports with regard to parasitic infections in mentally ill patients, studies focused on the association of mental illnesses with parasitic diseases are limited in the world. The public health significance of parasitic infections in these populations lies in the intricate interplay between mental health and physical well-being. Addressing parasitic infections in mentally ill patients is crucial as these infections can further compromise their overall health, exacerbate their mental health conditions, and lead to more challenging treatment courses. Moreover, the socio-economic and behavioral factors associated with mental illnesses, such as hygiene neglect, limited access to healthcare, and compromised immune responses, contribute to an increased risk of parasitic infections.

Therefore, in the current study, we aimed to conduct a comprehensive systematic review and meta-analysis to evaluate the worldwide status of infections caused by protozoan parasites in patients with mental health diseases. We further conducted a comprehensive investigation regarding the association between mental disorders and protozoan parasite infection.

## Methods

### Search strategy

The present study complies with the Preferred Reporting Items for Systematic Reviews and Meta-Analysis (PRISMA) checklist [18]. Multiple databases (PubMed, Scopus, Web of Science, ProQuest, and Google Scholar) were explored to obtain papers published from 2000 until December 2022 without a lower date limit. Search terms related to mental disorder, mental illness, mental disabilities, neurotic disorder, psychiatric disorders, psychiatric illness, protozoan parasites, protozoan pathogens, protozoan infections, protozoan diseases, prevalence, frequency, proportion, worldwide, global, using AND and/or OR Boolean operators. The duplicate papers were omitted automatically using the EndNote software X9 version. The references list was hand-searched to find further relevant studies that were not accessible through a database search. Two authors independently searched, evaluated titles and abstracts, and reviewed the full texts.

### Inclusion and exclusion criteria

Full-text articles were regarded eligible if they met the inclusion criteria described below:

1. Case–control and cross-sectional studies reporting the protozoan parasites among patients with mental disorders.
2. Peer-reviewed original articles.
3. Availability of full-text and abstract in English.
4. Availability of total sample size and the exact number of positive subjects.

Case series, case reports, letters, editorials, publications with non-original data, review articles, articles with unclear or ambiguous findings, non-English-language papers, and the papers that reported protozoan parasites in samples related to subjects other than humans were excluded from the analyses of the present study. Microsoft Excel^®^ version 2016 was employed to separately collect the following information from the included papers: author’s name, year of publication, annual precipitation, humidity, annual rainfall, average temperature, WHO region, income level, type of protozoan parasite, and diagnostic method (Tables 1, 2, 3, 4, 5, 6).

**Table 1:** Main characteristics of the included case–control studies reporting the prevalence of protozoan parasitic infections among patients with mental disorders

Study No	Author	Year	Study Years	Subjects type	Mental disorder	Continent	Diagnostic method	Case Size	Infected	Mean age	Control Size	Infected	Mean age	Species of parasites
1	Yolken et al.	2001	1998–1999	Hospitalized	Schizophrenia Spectrum and Other Psychotic Disorders	Europe	EIA & Western Blot	38	16	27	27	3	–	*Toxoplasma gondii*
2	Brown et al.	2005	1959–1967	Non hospitalized	Schizophrenia Spectrum and Other Psychotic Disorders	North America	Sabin-Feldman Dye Test	63	25	–	123	30	–	*Toxoplasma gondii*
3	El-Sahn et al.	2005	–	Non hospitalized	Schizophrenia Spectrum and Other Psychotic Disorders	Africa	EIA	75	60	–	85	45	–	*Toxoplasma gondii*
4	Alvarado-Esquivel et al.	2006	2005–2006	Hospitalized	Schizophrenia Spectrum and Other Psychotic Disorders	North America	ELISA	137	25	43.7	180	16	42	*Toxoplasma gondii*
5	Wang et al.	2006	–	Hospitalized/Non hospitalized	Schizophrenia Spectrum and Other Psychotic Disorders, Bipolar and Related Disorder	Asia	ELISA	1200	170	22.63	400	23	–	*Toxoplasma gondii*
6	Akyol et al.	2006	2003	Non hospitalized	Neurocognitive disorders	Asia	ELISA	100	31	28.88	50	10	27.56	*Toxoplasma gondii*
7	Mokhtari et al.	2006	–	Non hospitalized	Schizophrenia Spectrum and Other Psychotic Disorders	Asia	ELISA	230	41	–	230	17	–	*Toxoplasma gondii*
8	Cetinkaya et al.	2007	–	Non hospitalized	Schizophrenia Spectrum and Other Psychotic Disorders	Asia	ELISA	100	66	37.25	100	23	–	*Toxoplasma gondii*
9	Saraei-Sahnesaraei et al.	2007	–	Hospitalized	Schizophrenia Spectrum and Other Psychotic Disorders	Asia	ELISA	103	57	35.36	114	58	–	*Toxoplasma gondii*
10	Tamer et al.	2008	2004–2005	Hospitalized	Schizophrenia Spectrum and Other Psychotic Disorders	Asia	ELISA	40	16	–	37	5	–	*Toxoplasma gondii*
11	Yuksel et al.	2008	–	Non hospitalized	Schizophrenia and Other Psychotic Disorders	Asia	ELISA & Sabin-Feldman Dye Test	450	237	–	150	68	–	*Toxoplasma gondii*
12	Dogruman-Al et al	2009	2007–2008	Non hospitalized	Schizophrenia Spectrum and Other Psychotic Disorders	Asia	ELISA	88	42	38.5	88	19	29.2	*Toxoplasma gondii*
13	Xiao et al.	2009	–	Non hospitalized	Schizophrenia Spectrum and Other Psychotic Disorders	North America	ELISA	219	91	–	613	215	–	*Toxoplasma gondii*
14	Saraei-Sahnesaraei et al.	2009	–	Hospitalized	Schizophrenia Spectrum and Other Psychotic Disorders	Asia	ELISA	103	41	–	114	80	–	*Toxoplasma gondii*
15	Mahmoud et al.	2009	–	Hospitalized	Schizophrenia Spectrum and Other Psychotic Disorders	Asia	ELISA	96	55	38.25	96	22	–	*Toxoplasma gondii*
16	Daryani et al.	2010	2009	Hospitalized	Schizophrenia Spectrum and Other Psychotic Disorders	Asia	ELISA	80	55	32.95	91	61	–	*Toxoplasma gondii*
17	Miman et al.	2010	2008	Non hospitalized	Obsessive–Compulsive and Related Disorders	Asia	ELISA	42	20	34.05	100	19	38.1	*Toxoplasma gondii*
18	Tanyuksel et al.	2010	2002–2006	Non hospitalized	Schizophrenia Spectrum and Other Psychotic Disorders	Asia	ELISA & Sabin-Feldman Dye Test	70	32	23.4	40	15	30.3	*Toxoplasma gondii*
19	Xiao et al.	2010	2006–2008	Non hospitalized	Bipolar and Related Disorder, Trauma and Stressor Related Disorder, Schizophrenia Spectrum and Other Psychotic Disorders	Asia	ELISA	914	99	–	2634	328	–	*Toxoplasma gondii*
20	Yuksel et al.	2010	2007	Hospitalized	Obsessive–Compulsive and Related Disorders	Asia	ELISA	300	182	42.6	300	123	–	*Toxoplasma gondii*
21	Krause et al.	2010	–	Hospitalized	Schizophrenia Spectrum and Other Psychotic Disorders	Europe	ELISA	31	12	36.7	30	6	33.7	*Toxoplasma gondii*
22	Hamidinejat et al.	2010	–	Hospitalized/Non hospitalized	Schizophrenia Spectrum and Other Psychotic Disorders	Asia	ELISA	144	77	33	48	16	–	*Toxoplasma gondii*
23	Alipour et al.	2011	2009–2010	Hospitalized	Schizophrenia Spectrum and Other Psychotic Disorders	Asia	ELISA	62	42	37.54	62	23	37.24	*Toxoplasma gondii*
24	Alvarado-Esquivel et al.	2011	2009–2010	Hospitalized	Schizophrenia Spectrum and Other Psychotic Disorders	North America	ELISA	50	10	45.12	150	8	45.1	*Toxoplasma gondii*
25	Liu ET et al.	2011	–	Hospitalized	Bipolar and Related Disorder	Asia	ELISA	477	112	29.86	210	12	28.12	*Toxoplasma gondii*
26	Mortensen et al.	2011	1991–1994	Non hospitalized	Schizophrenia Spectrum and Other Psychotic Disorders	Europe	Optical density	127	33	–	127	26	–	*Toxoplasma gondii*
27	Bamne et al.	2012	–	Non hospitalized	Schizophrenia Spectrum and Other Psychotic Disorders	North America	ELISA	604	159	–	404	83	–	*Toxoplasma gondii*
28	Horacek et al.	2012	–	Non hospitalized	Schizophrenia Spectrum and Other Psychotic Disorders	Europe	ELISA & CFT	44	12	30.82	56	13	27.89	*Toxoplasma gondii*
29	Nascimento et al.	2012	–	Non hospitalized	Depressive Disorder, Schizophrenia Spectrum and Other Psychotic Disorders	South America	ELISA & ELFA	79	38	32.7	95	27	38.2	*Toxoplasma gondii*
30	Park et al.	2012	2010–2011	Hospitalized/Non hospitalized	Schizophrenia Spectrum and Other Psychotic Disorders	Asia	ELISA & IFA	96	21	46.14	50	4	44.8	*Toxoplasma gondii*
31	Emelia et al.	2012	2011	Non hospitalized	Schizophrenia Spectrum and Other Psychotic Disorders	Asia	ELISA	144	54	–	144	49	–	*Toxoplasma gondii*
32	El-Sayed et al.	2012	–	Hospitalized/Non hospitalized	Depressive Disorder, Schizophrenia Spectrum and Other Psychotic Disorders	Africa	ELISA	90	46	38.0	20	6	37.76	*Toxoplasma gondii*
33	James et al.	2013	2011–2014	Non hospitalized	Schizophrenia Spectrum and Other Psychotic Disorders	Africa	Rapid Test-Cassette	140	43	28.2	140	25	–	*Toxoplasma gondii*
34	Juanah et al.	2013	–	Hospitalized	Bipolar and Related Disorder	Asia	ELISA	88	45	–	88	27	–	*Toxoplasma gondii*
35	khademvatan et al.	2013	2011–2012	Hospitalized	Bipolar and Related Disorder	Asia	ELISA	117	37	33.93	200	53	33.88	*Toxoplasma gondii*
36	Pearce et al.	2013	–	Non hospitalized	Schizophrenia Spectrum and Other Psychotic Disorders	North America	ELISA	183	21	44.9	137	13	37.7	*Toxoplasma gondii*
37	Hamdani et al.	2013	–	Hospitalized	Schizophrenia Spectrum and Other Psychotic Disorders	Europe	ELISA	110	80	44.87	106	41	–	*Toxoplasma gondii*
38	Khademvatan et al.	2014	2011–2012	Hospitalized	Schizophrenia Spectrum and Other Psychotic Disorders	Asia	ELISA	100	34	36.39	200	53	25.04	*Toxoplasma gondii*
39	AL-Maamuri et al.	2014	2012–2013	Hospitalized	Schizophrenia Spectrum and Other Psychotic Disorders	Asia	ELISA & LAT	200	143	–	100	45	–	*Toxoplasma gondii*
40	Ebadi et al.	2014	2011–2012	Non hospitalized	Schizophrenia Spectrum and Other Psychotic Disorders	Asia	ELISA	152	129	32.7	152	93	33.5	*Toxoplasma gondii*
41	Elsaid et al.	2014	–	Hospitalized/Non hospitalized	Schizophrenia Spectrum and Other Psychotic Disorders	Africa	ELISA	300	151	–	300	66	–	*Toxoplasma gondii*
42	Karabulut et al.	2015	–	Hospitalized	Schizophrenia Spectrum and Other Psychotic Disorders	Asia	ELISA	85	35	41.73	60	26	40.45	*Toxoplasma gondii*
43	Omar et al.	2015	–	Hospitalized	Schizophrenia Spectrum and Other Psychotic Disorders	Asia	ELISA & PCR	101	52	41.1	55	10	45.3	*Toxoplasma gondii*
44	Cong et al.	2015	2011–2013	Non hospitalized	Neurocognitive disorders	Asia	ELISA	445	77	–	445	55	–	*Toxoplasma gondii*
45	Khattak et al.	2015	2013–2014	Hospitalized	Schizophrenia Spectrum and Other Psychotic Disorders	Asia	ELISA	142	58	8.61	76	12	8.42	*Toxoplasma gondii*
46	Cevizci et al.	2015	–	Hospitalized	Schizophrenia Spectrum and Other Psychotic Disorders	Asia	ELISA	30	10	–	60	13	–	*Toxoplasma gondii*
47	Bakre et al.	2015	–	Hospitalized	Neurodevelopmental Disorders	Asia	ELISA & LAT	93	41	–	93	12	–	*Toxoplasma gondii*
48	Alvarado-Esquivel et al.	2015	2013–2014	Non hospitalized	Neurocognitive disorders	North America	ELISA	149	15	36.01	149	14	36.03	*Toxoplasma gondii*
49	Esshili et al.	2016	–	Hospitalized	Bipolar and Related Disorder	Africa	ELISA	246	184	40.5	117	63	38.6	*Toxoplasma gondii*
50	Kheirandish et al.	2016	2015	Non hospitalized	Bipolar and Related Disorder, Schizophrenia Spectrum and Other Psychotic Disorders	Asia	ELISA	170	103	–	170	65	–	*Toxoplasma gondii*
51	Zaki et al.	2016	–	Hospitalized	Neurodevelopmental Disorders	Asia	ELISA	162	58	–	163	24	–	*Toxoplasma gondii*
52	Menati Rashno et al.	2016	2014–2015	Non hospitalized	Schizophrenia Spectrum and Other Psychotic Disorders	Asia	ELISA	87	58	78.17	87	49	45.63	*Toxoplasma gondii*
53	El-Aal et al.	2016	2015–2016	Non hospitalized	Neurodevelopmental Disorders and Other Psychotic Disorders	Africa	ELISA	230	50	–	60	7	–	*Toxoplasma gondii*
54	Youssef Saad et al.	2016	2015–2016	Non hospitalized	Schizophrenia Spectrum and Other Psychotic Disorders	Africa	ELISA & Western Blot	100	52		100	11		*Toxoplasma gondii*
55	Dalimiasl et al.	2016	–	Non hospitalized	Schizophrenia Spectrum and Other Psychotic Disorders	Asia	ELISA	76	42	–	75	27	–	*Toxoplasma gondii*
56	Abdollahian et al.	2017	2011–2012	Hospitalized	Schizophrenia Spectrum and Other Psychotic Disorders	Asia	ELISA	350	164	35.0	350	120	38.0	*Toxoplasma gondii*
57	Alvarado-Esquivel et al.	2017	2014	Non hospitalized	Schizophrenia Spectrum and Other Psychotic Disorders	North America	ELISA & PCR	65	6	40.3	195	21	40.6	*Toxoplasma gondii*
58	Ansari-Lari et al.	2017	–	Non hospitalized	Depressive Disorder	Asia	ELISA	99	42	40.3	152	41	40.6	*Toxoplasma gondii*
59	Bak et al.	2017	2015–2016	Non hospitalized	Obsessive–Compulsive and Related Disorders	Asia	ELISA	155	21	43.75	135	8	41.59	*Toxoplasma gondii*
60	Akaltun et al.	2017	–	Non hospitalized	Neurocognitive disorders	Asia	ELISA & Sabin-Feldman Dye Test	120	40	–	60	6	–	*Toxoplasma gondii*
61	Fallahi et al.	2017	–	Non hospitalized	Neurocognitive disorders	Asia	ELISA & PCR	115	61	75.2	115	64	74.1	*Toxoplasma gondii*
62	Rashno et al.	2017	–	Non hospitalized	Schizophrenia Spectrum and Other Psychotic Disorders	Asia	ELISA	87	46	–	87	35	–	*Toxoplasma gondii*
63	Campos-Carli et al.	2017	–	Non hospitalized	Neurodevelopmental Disorders	South America	ELISA	40	27	40.62	48	32	40.21	*Toxoplasma gondii*
64	Hamed et al.	2018	2017–2018	Non hospitalized	Neurodevelopmental Disorders	Africa	IHAT	200	84	41.15	200	35	35.58	*Toxoplasma gondii*
65	Stepanova et al.	2018	2016	Non hospitalized	Schizophrenia Spectrum and Other Psychotic Disorders	Europe	ELISA	115	62	–	152	39	–	*Toxoplasma gondii*
66	Muflikhah et al.	2018	2015	Non hospitalized	Schizophrenia Spectrum and Other Psychotic Disorders	Asia	ELISA	94	65	–	64	42	–	*Toxoplasma gondii*
67	Wokem et al.	2018	–	Hospitalized	Bipolar and Related Disorder	Africa	ELISA	200	109	–	200	57	–	*Toxoplasma gondii*
68	Alvarado-Esquivel	2019	–	Hospitalized	Schizophrenia Spectrum and Other Psychotic Disorders	North America	EIA	66	6	–	396	22	–	*Toxoplasma gondii*
69	Chen et al.	2019	2016–2018	Hospitalized	Neurodevelopmental Disorders	Asia	ECLIA	798	106	38.83	681	64	37.66	*Toxoplasma gondii*
70	Fentahun et al.	2019	2018	Hospitalized	Depressive Disorder	Africa	Direct smear & Sedimentation	104	21	14.05	314	49	11.96	*Giardia lamblia & Entamoeba histolytica/Entamoeba dispar*
71	Sapmaz et al.	2019	2017–2018	Non hospitalized	Schizophrenia Spectrum and Other Psychotic Disorders	Asia	ELISA	37	8	15.6	36	2	14.55	*Toxoplasma gondii*
72	Stepanova et al.	2019	2016	Non hospitalized	Schizophrenia Spectrum and Other Psychotic Disorders	Europe	ELISA	155	62	–	159	32	–	*Toxoplasma gondii*
73	Achaw et al.	2019	2018	Hospitalized	Schizophrenia Spectrum and Other Psychotic Disorders	Africa	Rapid Test-Cassette	152	53	–	152	31	–	*Toxoplasma gondii*
74	Alshehri et al.	2019	–	Non hospitalized	Schizophrenia Spectrum and Other Psychotic Disorders	Asia	ELISA	30	18	–	20	6	–	*Toxoplasma gondii*
75	Oana et al.	2019	2011–2012	Hospitalized	Schizophrenia Spectrum and Other Psychotic Disorders	Europe	ELISA	91	40	38.7	206	73	48.9	*Toxoplasma gondii*
76	El-Gebaly et al.	2019	2018	Hospitalized	Depressive Disorder	Africa	ELISA	120	54	–	120	59	–	*Toxoplasma gondii*
77	Nasirpour et al.	2020	–	Non hospitalized	Schizophrenia Spectrum and Other Psychotic Disorders	Asia	ELISA & PCR	87	53	61.9	87	49	61.9	*Toxoplasma gondii*
78	Ali et al.	2020	2018–2019	Non hospitalized	Schizophrenia Spectrum and Other Psychotic Disorders	Africa	ELISA & Western Blot	45	25	39.82	45	13	37.35	*Toxoplasma gondii*
79	Huseein et al.	2020	2016	Hospitalized	Schizophrenia Spectrum and Other Psychotic Disorders	Africa	ELISA	110	57	–	50	15	–	*Toxoplasma gondii*
80	Kezai et al.	2020	–	Hospitalized	Schizophrenia Spectrum and Other Psychotic Disorders	Africa	ELISA	70	49	40.76	70	37	37.97	*Toxoplasma gondii*
81	Al-Antably et al.	2021	–	Non hospitalized	Depressive Disorder	Africa	ELISA	150	64	–	150	29	–	*Toxoplasma gondii*
82	Bahceci et al.	2021	–	Non hospitalized	Depressive Disorder, Schizophrenia Spectrum and Other Psychotic Disorders	Asia	ELISA	200	72	–	100	21	29.73	*Toxoplasma gondii*
83	Ekici et al.	2021	2018–2020	Non hospitalized	Schizophrenia Spectrum and Other Psychotic Disorders	Asia	ELISA	190	120	–	100	29	–	*Toxoplasma gondii*
84	Kamal et al.	2021	2017–2019	Non hospitalized	Bipolar and Related Disorder	Africa	Direct smear & Sedimentation & Stain	983	337	36.2	983	105	35.4	*Giardia lamblia & Blastocystis hominis &E. histolytica dispar & Entamoeba coli & Cryptosporidium spp & Cyclospora cayetanensis*
85	sirin et al.	2021	–	Non hospitalized	Schizophrenia Spectrum and Other Psychotic Disorders	Asia	ECLIA	48	9	–	50	10	–	*Toxoplasma gondii*
86	Zahariluddin et al.	2021	–	Non hospitalized	Obsessive–Compulsive and Related Disorders	Asia	ELISA	109	26	–	109	35	–	*Toxoplasma gondii*
87	sirin et al.	2021	2019–2020	Non hospitalized	Schizophrenia Spectrum and Other Psychotic Disorders	Asia	ECLIA	38	6	37.13	48	9	36.08	*Toxoplasma gondii*
88	Grada et al.	2022	2018–2019	Hospitalized	Schizophrenia Spectrum and Other Psychotic Disorders	Europe	ECLIA	308	209	45.64	296	160	45.29	*Toxoplasma gondii*
89	Liu et al.	2022	2015–2020	Hospitalized	Schizophrenia Spectrum and Other Psychotic Disorders	Asia	ELISA	3101	94	32.85	2194	23	32.33	*Toxoplasma gondii*
90	Mohammed	2022	–	Hospitalized	Schizophrenia Spectrum and Other Psychotic Disorders	Asia	ELISA & LAT	45	28	–	40	13	–	*Toxoplasma gondii*
91	Ademe et al.	2022	2018–2019	Hospitalized	Schizophrenia Spectrum and Other Psychotic Disorders	Africa	ECLIA	47	41	29.64	47	38	30.98	*Toxoplasma gondii*

*EIA* Enzyme Immunoassay, *SFDT* Sabin-Feldman Dye Test, *ELISA* Enzyme-Linked Immunosorbent Assay, *CFT* Complement fixation test, *ELFA* Enzyme-linked flourescence assay, *IFA* Immunofluorescence assay, *IHAT* Indirect haemagglutination test, *ECLIA* Electrochemiluminiscence immunoassay, *CLIA* Chemiluminescent immunoassay

**Table 2:** Sub-group analysis based on annual precipitation, humidity, annual rainfall, average temperature, WHO regions, income level, and diagnostic method in included case–control studies

Variables	No studies	Sample size	Infected	Sample size (control)	Infected (control)	Pooled OR (95% CI)	Heterogeneity		
							*I2*	τ^2^	*p*-value
Annual precipitation									
< 300	62	14,961	4533	13,714	2681	2.130 (1.844–2.460)	77	0245	*p* < 0.001
300–650	8	1024	394	995	221	2.297 (1.700- 3.103)	32	0.087	*p* < 0.001
651–1000	13	1842	499	2637	498	1.827 (1.323–2.522)	54	0.210	*p* < 0.001
> 1000	8	839	357	973	281	1.579 (1.042–2.392)	66	0.245	*p* < 0.001
Total	91	18,666	5783	18,319	3681	2.059 (1.830–2.317)	74	0.247	*p* < 0.001
Humidity (%)									
< 40	19	2354	1161	2524	934	1.749 (1.342–2.279)	74	0.272	*p* < 0.001
40–75	66	15,471	4196	14,897	2393	2.194 (1.917–2.510)	73	0.228	*p* < 0.001
> 75	6	841	426	898	354	1.629 (1.971–2.734)	74	0.337	*p* < 0.001
Total	91	18,666	5783	18,319	3681	2.059 (1.830–2.317)	74	0.247	*p* < 0.001
Annual rainfall (mm)									
< 400	35	5261	2457	5036	1454	2.256 (1.848–2.761)	79	0.293	*p* < 0.001
401–1000	37	10,743	2402	10,309	1439	2.040 (1.699–2.449)	72	0.251	*p* < 0.001
1001–1500	14	2126	682	2514	625	1.773 (1.444–2.178)	28	0.060	*p* < 0.001
> 1500	5	536	242	460	163	1.458 (0.803–2.985)	78	0.455	*p* < 0.001
Total	91	18,666	5783	18,319	3681	2.059 (1.830–2.317)	74	0.247	*p* < 0.001
Average temperature (°C)									
< 10	5	550	197	551	119	2.036 (1.319–3.144)	22	0.143	*p* < 0.001
10–20	53	13,544	3787	12,801	2589	1.947 (1.669–2.270)	74	0.266	*p* < 0.001
> 20	33	4572	1799	4967	973	2.281 (1.8507–2.811)	72	0.237	*p* < 0.001
Total	91	18,666	5783	18,319	3681	2.059 (1.830–2.317)	74	0.247	*p* < 0.001
WHO region									
African region	7	959	500	1040	300	2.175 (1.697–2.778)	0	0.029	*p* < 0.001
Eastern Mediterranean Region	35	5333	2466	4992	1421	2.289 (1.868–2.805)	79	0.299	*p* < 0.001
European Region	26	3984	1492	5253	1099	1.844 (1.477–2.301)	71	0.259	*p* < 0.001
Region of the Americas	11	1582	482	2459	509	1.816 (1.330–2.479)	55	0.175	*p* < 0.001
Western Pacific Region	11	6714	778	4511	310	2.157 (1.535–3.032)	75	0.268	*p* < 0.001
South-East Asia Region	1	94	65	64	42	1.174 (0.596–2.309)	–	–	–
Total	91	18,666	5783	18,319	3681	2.059 (1.830–2.317)	74	0.247	*p* < 0.001
Income level									
High income	16	2260	732	2330	580	2.008 (1.483–2.718)	68	0.248	*p* < 0.001
Upper middle income	36	10,606	2312	10,389	1468	1.930 (1.614–2.308)	72	0.238	*p* < 0.001
Lower middle income	39	5800	2739	5600	1633	2.190 (1.823–2.632)	77	0.263	*p* < 0.001
Total	91	18,666	5783	18,319	3681	2.059 (1.830–2.317)	74	0.247	*p* < 0.001
Diagnostic method									
ELISA	61	13,769	3893	13,369	2658	2.010 (1.752–2.307)	74	0.236	*p* < 0.001
IHAT	1	200	84	200	35	3.413 (2.154–5.409)	–	–	–
ELISA & Western Blot	2	145	77	145	24	3.076 (1.285–7.362)	–	–	–
EIA & Western blotting	1	38	16	27	3	5.818 (1.490–22.715)	–	–	–
EIA	2	141	66	481	67	2.617 (1.241–5.515)	33	0.116	*p* < 0.001
Sabin-Feldman Dye Test	2	133	57	163	45	1.427 (0.667–3.054)	55	0.164	*p* < 0.001
ELISA & CFT	1	44	12	56	13	1.240 (0.500- 3.076)	–	–	–
ELISA & ELFA	1	79	38	95	27	2.230 (1.334–4.103)	–	–	–
ELISA & IFA	1	96	21	50	4	3.220 (1.039–9.973)	–	–	–
Rapid Test-Cassette	2	292	96	292	56	2.066 (1.412- 3.022)	–	–	–
ELISA & PCR	4	368	172	452	144	1.432 (0.656–1.123)	77	0.498	*p* < 0.001
Direct smear & Sedimentation	1	104	21	314	49	1.368 (0.775–2.413)	–	–	–
Direct smear & Sedimentation & Stain	1	983	337	983	105	4.362 (3.426–5.553)	–	–	–
Optical density	1	127	33	127	26	1.363 (0.759–2.449)	–	–	–
ECLIA	5	1239	371	1122	281	1.470 (1.064–2.032)	0	0.042	*p* < 0.001
ELISA & LAT	3	338	212	233	70	3.147 (2.033–4.869)	0	0.0001	*p* < 0.001
ELISA & Sabin-Feldman Dye Test	2	570	277	210	74	2.258 (0.734–6.947)	82	0.541	*p* < 0.001
Total	91	18,666	5783	18,319	3681	2.059 (1.830–2.317)	74	0.247	*p* < 0.001

_*EIA* Enzyme Immunoassay, *SFDT* Sabin-Feldman Dye Test, *ELISA* Enzyme-Linked Immunosorbent Assay, *CFT* Complement fixation test, *ELFA* Enzyme-linked fluorescence assay, *IFA* Immunofluorescence assay, *IHAT* Indirect haemagglutination test, *ECLIA* Electrochemiluminiscence immunoassay, *CLIA* Chemiluminescent immunoassay_

**Table 3:** Sub-group analysis based on type of protozoan parasites in case–control studies

Type of intestinal protozoan parasites	No. studies	Sample size-case	Infected case	Sample size-control	Infected control	Pooled OR (95% CI)	Heterogeneity		
							*I*^2^	τ^2^	*p*-value
*Cyclospora cayetanensis*	1	983	14	983	3	4.719 (1.352–16.474)	–	–	–
*Cryptosporidium parvum*	1	983	94	983	22	4.618 (2.877–7.412)	–	–	–
*Blastocystis hominis*	1	983	157	983	51	3.473 (2.498–4.829)	–	–	–
*Giardia lamblia*	2	1987	25	1297	18	2.461 (0.718–8.429)	67	0.511	*p* < 0.001
*Entamoeba histolytica /dispar*	2	1087	52	1297	44	2.350 (0.802–6.888)	80	0.477	*p* < 0.001
*Toxoplasma gondii*	89	17,579	5425	17,022	3527	2.037 (1.808–2.294)	71	0.246	*p* < 0.001
*Entamoeba coli*	1	983	10	983	16	0.621 (0.280–1.375)	–	–	–

**Table 4:** Main characteristics of the included cross-sectional studies reporting the prevalence of protozoan parasitic infections among patients with mental disorders

Study No	Author	Year	Study Years	Subjects type	Mental disorder	Continent	Diagnostic method	F/M	Mean age	Sample size	Infected	Species of parasites
**1**	Gatti et al.	2000	–	Hospitalized	Schizophrenia Spectrum and Other Psychotic Disorders	Europe	Direct smear & ELISA & Culture	–	44.0	550	70	*Entamoeba histolytica/Entamoeba dispar &* *Entamoeba coli &* *Endolimax nana &* *Blastocystis hominis &* *Giardia lamblia*
**2**	Mahyar et al.	2000	–	Non Hospitalized	Neurodevelopmental Disorders	Asia	Direct smear & Sedimentation	–	–	258	98	*Entamoeba histolytica & Giardia lamblia &* *Idomoeba butschlii & Entamoeba coli*
**3**	Thomas et al.	2004	1998–1999	Non Hospitalized	Schizophrenia Spectrum and Other Psychotic Disorders	Europe	ELISA	–	–	370	166	*Toxoplasma gondii*
**4**	Cheng et al.	2005	–	Hospitalized	Schizophrenia Spectrum and Other Psychotic Disorders	Asia	Direct smear & Sedimentation	–	41.0	464	10	*Entamoeba histolytica & Entamoeba coli*
**5**	Gharavi et al.	2005		Hospitalized	Neurodevelopmental Disorders	Asia	IFA	–	–	353	49	*Toxoplasma gondii*
**6**	Rivera et al.	2006		Non Hospitalized	Neurodevelopmental Disorders	Asia	Direct smear & PCR	–	–	113	80	*Giardia lamblia & Entamoeba histolytica & Entamoeba coli &* *Endolimax nana &* *Blastocystis hominis*
**7**	Dickerson et al.	2007	–	Non Hospitalized	Schizophrenia Spectrum and Other Psychotic Disorders	North America	Solid-phase immunoassay	–	40.5	358	58	*Toxoplasma gondii*
**8**	Sharif et al.	2007	2004–2005	Hospitalized	Neurodevelopmental Disorders	Asia	IFA	175/161	–	336	140/125	*Toxoplasma gondii*
**9**	Hazrati Tappeh et al.	2010	–	Hospitalized	Neurodevelopmental Disorders	Asia	Direct smear & Sedimentation	–	–	225	39	*Entamoeba histolytica/Entamoeba dispar*
**10**	Sharif et al.	2010	2008	Hospitalized	Neurodevelopmental Disorders	Asia	Direct smear & Sedimentation	–	25.48	362	77	*Entamoeba histolytica & Giardia lamblia &* *Blastocystis hominis & Entamoeba coli & Endolimax nana*
**11**	Chandrasena et al.	2010	2009	Non Hospitalized	Bipolar and Related Disorder	Asia	Direct smear	–	50	145	3	*Entamoeba coli*
**12**	Pearce et al.	2012	1988–1994	Non Hospitalized	Depressive Disorder	North America	IFA & Sabin-Feldman dye test	–	-	7774	1211	*Toxoplasma gondii*
**13**	Shokri et al.	2012		Hospitalized	Neurodevelopmental Disorders	Asia	Direct smear & Sedimentation& Stain	–	22.8	133	30	*Giardia lamblia & Blastocystis hominis & Entamoeba coli & chilomastix mesnil*
**14**	Khalili et al.	2013	2010	Hospitalized	Schizophrenia Spectrum and Other Psychotic Disorders	Asia	Direct smear & Sedimentation& Stain	–	41.2	65	31	*Giardia lamblia & Blastocystis hominis & Entamoeba coli & Cryptosporidium spp &* *Idomoeba butschlii &* *Endolimax nana & Cystoisospora belli*
**15**	Anvari et al.	2015	–	Hospitalized	Neurodevelopmental Disorders	Asia	Direct smear & Sedimentation& Stain	–	–	129	55	*Blastocystis hominis & Entamoeba coli & Endolimax nana & chilomastix mesnil & Idomoeba butschlii & Giardia lamblia*
**16**	Duffy et al.	2015	2011–2013	Non Hospitalized	Depressive Disorder	North America	ELISA	–	47.0	70	8	*Toxoplasma gondii*
**17**	Ahmadi et al.	2015	2013–2014	Hospitalized	Neurodevelopmental Disorders	Asia	Direct smear & Culture & Stain	–	–	341	67	*Idomoeba butschlii & Entamoeba coli & chilomastix mesnil*
**18**	Ezatpour et al.	2015	2012–2013	Hospitalized	Neurodevelopmental Disorders	Asia	ELISA	64/94	–	158	18/30	*Toxoplasma gondii*
**19**	Fond et al.	2015	2019–2011	Hospitalized & Non-Hospitalized	Schizophrenia Spectrum, Other Psychotic Disorders, Bipolar and Related Disorder	Europe	ELISA	–	–	266	162	*Toxoplasma gondii*
**20**	Shehata et al.	2015	2012–2013	Non Hospitalized	Neurodevelopmental Disorders	Africa	Direct smear & Sedimentation& Stain	78/122	–	200	30/57	*Giardia lamblia & Blastocystis hominis & Entamoeba histolytica & Entamoeba coli Cyclospora cayetanensis & Dientamoeba fragilis*
**21**	Saeidinia et al.	2016	2013	Non-Hospitalized	Neurodevelopmental Disorders	Asia	Direct smear & Sedimentation & Culture	–	25.69	173	41	*Entamoeba coli & Endolimax nana & Giardia lamblia & Blastocystis hominis*
**22**	Soleymani et al.	2016	2014	Hospitalized	Neurodevelopmental Disorders	Asia	Direct smear & Willis	–	35.0	97	4	*Blastocystis hominis & Entamoeba coli*
**23**	Sugden et al.	2016	1972–1973	Non-Hospitalized	Depressive Disorder, Schizophrenia Spectrum, Other Psychotic Disorders	Oceania	EIA	–	38.0	472	43	*Toxoplasma gondii*
**24**	Shehata et al.	2016	2014–2015	Non-Hospitalized	Neurodevelopmental Disorders	Africa	ELISA	74/114	16.84	188	52/73	
**25**	Freitas et al.	2017	–	Hospitalized	Neurocognitive Disorders	South America	Ritchie's modified method & Lutz	–	–	156	13	*Blastocystis hominis & Entamoeba coli & Entamoeba histolytica/Entamoeba dispar & Giardia lamblia & Endolimax nana*
**26**	Massa et al.	2017	–	Non-Hospitalized	Depressive Disorder, Trauma, Stressor Related Disorder, Schizophrenia Spectrum and Other Psychotic Disorders	North America	ELISA	–	–	717	97	*Toxoplasma gondii*
**27**	Nyundo et al.	2017	–	Hospitalized	Schizophrenia Spectrum and Other Psychotic Disorders	Africa	Direct smear & Sedimentation	–	32.7	363	3	*Entamoeba histolytica/Entamoeba dispar*
**28**	Olariu et al.	2017	2011–2012	Non-Hospitalized	Schizophrenia Spectrum and Other Psychotic Disorders	Europe	ELISA	–	–	214	117	*Toxoplasma gondii*
**29**	Fond et al.	2018	–	Non-Hospitalized	Schizophrenia Spectrum and Other Psychotic Disorders	Europe	ELISA	–	–	289	75	*Toxoplasma gondii*
**30**	Jafari Modrek et al	2018	2016	Non-Hospitalized	Schizophrenia Spectrum and Other Psychotic Disorders	Asia	ELISA & PCR	–	–	118	41	*Toxoplasma gondii*
**31**	Mohammadi-Meskin et al.	2019	2016–2017	Hospitalized	Neurodevelopmental Disorders	Asia	Direct smear & Sedimentation& Stain	–	25.9	126	79	*Blastocystis hominis & Entamoeba coli & Idomoeba butschlii & Giardia lamblia*
**32**	Eze et al.	2019	2017	Hospitalized & Non-Hospitalized	Schizophrenia Spectrum and Other Psychotic Disorders	Africa	Direct smear & Sedimentation	109/94	42.5	203	10/11	*Giardia lamblia & Entamoeba histolytica/Entamoeba dispar*
**33**	Otu-Bassey et al.	2019	2016	Hospitalized	Neurodevelopmental Disorders	Africa	Direct smear & Sedimentation		38.02	126	36	*Giardia lamblia & Entamoeba histolytica/Entamoeba dispar*
**34**	Galvan-Ramirez	2021	2019	Hospitalized	Schizophrenia Spectrum and Other Psychotic Disorders	South America	ELISA	12/15	38.3	27	14	*Toxoplasma gondii*
**35**	Matini et al.	2021	2017	Non-Hospitalized	Neurodevelopmental Disorders	Asia	Direct smear & Sedimentation& Stain	–	–	318	87	*Dientamoeba fragilis & Blastocystis hominis & Entamoeba coli & Idomoeba butschlii & Giardia lamblia*
**36**	Agmas et al.	2021	2020	Non-Hospitalized	Schizophrenia Spectrum and Other Psychotic Disorders	Africa	Direct smear & Sedimentation& Stain	–	34.2	432	63	*Giardia lamblia & Entamoeba histolytica/Entamoeba dispar*
**37**	Pakmehr et al.	2022	2021	Hospitalized	Neurodevelopmental Disorders	Asia	Direct smear & Sedimentation& Stain	64/55	27.6	119	15/16	*Giardia lamblia & Blastocystis hominis & Entamoeba coli & Idomoeba butschlii & Endolimax nana*
**38**	Liu et al.	2022	2015–2020	Hospitalized	Schizophrenia Spectrum and Other Psychotic Disorders	Asia	ELISA	–	–	3101	101	*Toxoplasma gondii*
**39**	Mohammed et al.	2022	2021–2022	Hospitalized	Schizophrenia Spectrum and Other Psychotic Disorders	Asia	ELISA	31/70	40.52	101	40	*Toxoplasma gondii*
**40**	Teimouri et al.	2022	2021	Hospitalized	Schizophrenia Spectrum and Other Psychotic Disorders	Asia	ELISA	135/183	35.91	318	80	*Toxoplasma gondii*

*ELISA* Enzyme-Linked Immunosorbent Assay, *IFA* Immunofluorescence assay, *SFDT* Sabin-Feldman Dye Test, *EIA* Enzyme Immunoassay

**Table 5:** Sub-group analysis based on annual precipitation, humidity, annual rainfall, average temperature, WHO regions, income level, mean age, and diagnostic method in included cross-sectional studies

Variables	No studies	Sample size	Infected	Pooled prevalence (95% CI)	Heterogeneity		
					*I*^*2*^	τ^2^	*p*-value
Annual precipitation							
< 300	27	8820	1720	0.270 (0.192–0.355)	98	0.057	*p* < 0.001
300–650	2	526	179	0.243 (0.006–0.655)	98	0.093	*p* < 0.001
651–1000	8	9639	1668	0.234 (0.119–0.372)	97	0.049	*p* < 0.001
> 1000	3	1009	153	0.239 (0.000–0713)	99	0.188	*p* < 0.001
Total	40	19,994	3720	0.252 (0.189–0.320)	98	0.063	*p* < 0.001
Humidity (%)							
< 40	17	3629	1117	0.303 (0.218–0.394)	97	0.039	*p* < 0.001
40–75	16	14,224	2181	0.240 (0.151–0.342)	98	0.050	*p* < 0.001
> 75	7	2141	422	0.201 (0.033–0.459)	99	0.139	*p* < 0.001
Total	40	19,994	3720	0.252 (0.189–0.320)	98	0.063	*p* < 0.001
Annual rainfall (mm)							
< 400	20	4118	1369	0.331 (0.249–0.418)	97	0.040	*p* < 0.001
401–1000	6	4447	539	0.315 (0.125–0.544)	99	0.081	*p* < 0.001
1001–1500	11	10,707	1719	0.142 (0.082–0.215)	97	0.024	*p* < 0.001
> 1500	3	722	93	0.174 (0.000–0.694)	99	0.235	*p* < 0.001
Total	40	19,994	3720	0.252 (0.189–0.320)	98	0.063	*p* < 0.001
Average temperature (°C)							
< 10	1	370	166	0.448 (0.398–0.499)	–	–	–
10–20	27	17,106	3059	0.263 (0.101–0.403)	98	0.044	*p* < 0.001
> 20	12	2518	495	0.0235 (0.018- 0.106)	98	0.099	*p* < 0.001
Total	40	19,994	3720	0.252 (0.189–0.320)	98	0.063	*p* < 0.001
WHO region							
African region	4	1124	123	0.011 (0.0001–0.368)	97	0.035	*p* < 0.001
Eastern Mediterranean Region	20	4118	1369	0.331 (0.244–0.424)	97	0.040	*p* < 0.001
European Region	5	1689	590	0.386 (0.150–0.656)	98	0.047	*p* < 0.001
Region of the Americas	6	8768	1401	0.170 (0.059–0.322)	83	0.025	*p* < 0.001
Western Pacific Region	4	4150	234	0.156 (0.000–0.744)	99	0.156	*p* < 0.001
South-East Asia Region	1	145	3	0.020 (0.007–0.059)	–	–	–
Total	40	19,994	3720	0.252 (0.189–0.320)	98	0.063	*p* < 0.001
Income level							
High income	9	10,532	1890	0.219 (0.122–0.336)	98	0.038	*p* < 0.001
Upper middle income	6	4063	295	0.215 (0.049–0.452)	98	0.097	*p* < 0.001
Lower middle income	25	5399	1535	0.285 (0.201–0.378)	98	0.060	*p* < 0.001
Total	40	19,994	3720	0.252 (0.189–0.320)	98	0.063	*p* < 0.001
Mean age							
15–20	1	188	125	0.664 (0.594–0.724)	–	–	–
20–25	4	794	227	0.316 (0.146–0.516)	96	0.041	*p* < 0.001
26–30	1	119	31	0.260 (0.190–0.346)	–	–	–
31–35	4	1210	150	0.090 (0.013–0.224)	97	0.034	*p* < 0.001
36–40	4	975	156	0.277 (0.135–0.445)	95	0.036	*p* < 0.001
41–45	5	1394	268	0.250 (0.039–0.558)	99	0.103	*p* < 0.001
46–50	2	215	11	0.056 (0.0004–0.174)	86	0.061	*p* < 0.001
Total	21	4895	968	0.252 (0.189–0.320)	98	0.063	*p* < 0.001
Diagnostic method							
Direct smear & Sedimentation	7	2001	284	0.141 (0.051–0.265)	98	0.042	*p* < 0.001
Direct smear & Sedimentation & Stain	8	1522	463	0.349 (0.241–0.465)	95	0.026	*p* < 0.001
Direct smear & PCR	1	113	80	0.708 (0.618–0.783)	–	–	–
Direct smear	1	145	3	0.020 (0.007–0.059)	–	–	–
ELISA	12	5819	1033	0.335 (0.214–0.468)	99	0.055	*p* < 0.001
Direct smear & ELISA & Culture	1	550	70	0.127 (0.102–0.157)	–	–	–
IFA	2	689	309	0.443 (0.001–0.974)	99	0.237	*p* < 0.001
Solid-phase immunoassay	1	358	58	0.162 (0.127–0.203)	–	–	–
IFA & Sabin-Feldman dye test	1	7440	1211	0.162 (0.154–0.171)	–	–	–
Direct smear & Culture & Stain	1	341	67	0.196 (0.157–0.241)	–	–	–
Direct smear & Sedimentation & Culture	1	173	41	0.237 (0.179–0.305)	–	–	–
Direct smear & Willis	1	97	4	0.041 (0.016–0.101)	–	–	–
EIA	1	472	43	0.091 (0.068–0.120)	–	–	–
ELISA & PCR	1	118	41	0.347 (0.267–0.437)	–	–	–
Ritchie's modified method & Lutz	1	156	13	0.083 (0.049–0.137)	–	–	–
Total	40	19,994	3720	0.252 (0.189–0.320)	98	0.063	*p* < 0.001

*ELISA* Enzyme-Linked Immunosorbent Assay, *IFA* Immunofluorescence assay, *SFDT* Sabin-Feldman Dye Test, *EIA* Enzyme Immunoassay

**Table 6:** Sub-group analysis based on type of protozoan parasites in cross-sectional studies

Type of intestinal protozoan parasites	No studies	Sample size	Infected	Pooled prevalence (95% CI)	Heterogeneity		
					*I*^2^	τ^2^	*p*-value
*Toxoplasma gondii*	17	13,849	2616	0.343 (0.228–0.467)	99	0.064	*p* < 0.001
*Cryptosporidium* spp.	3	384	53	0.087 (0.005–0.242)	95	0.032	*p* < 0.001
*Blastocystis hominis*	14	2766	224	0.085 (0.477–0.132)	93	0.018	*p* < 0.001
*Entamoeba coli*	18	3974	334	0.083 (0.050–0.123)	92	0.018	*p* < 0.001
*Cystoisospora belli*	1	65	5	0.076 (0.224–0.156)	–	–	–
*Cyclospora cayetanensis*	1	200	15	0.075 (0.042–0.116)	–	–	–
*Entamoeba histolytica/ dispar*	13	3870	251	0.064 (0.011–0.151)	97	0.067	*p* < 0.001
*Giardia lamblia*	17	3688	246	0.062 (0.040–0.088)	89	0.008	*p* < 0.001
*Endolimax nana*	10	2118	60	0.029 (0.006–0.067)	88	0.017	*p* < 0.001
*Dientamoeba fragilis*	2	518	17	0.029 (0.000–1.000)	95	0.023	*p* < 0.001
*Iodamoeba butschlii*	8	1581	43	0.024 (0.013–0.037)	53	0.001	*p* < 0.001
*Chilomastix mesnili*	3	603	13	0.021 (0.001–0.058)	77	0.005	*p* < 0.001

### Quality assessment

A Newcastle–Ottawa Scale was applied to assess the study quality (Additional file 1: Table S1 and Additional file 2: Table S2) [19, 20]. Scoring was according to the items and score ranges as follows: (1) Selection (maximum of 5 stars), (2) Comparability (maximum of 2 stars), and (3) Outcome (maximum of 3 stars).

### Data synthesis and statistical analysis

The overall pooled odds ratio (OR), and pooled prevalence reporting on protozoan parasites among patients with mental disorders at the global scale were calculated with a 95% confidence interval (95% CI). A Freeman-Tukey double arcsine transformation for the random-effects model was used to estimate the pooled prevalence. Begg’s rank test was applied to specify the possible publication bias. Furthermore, publication bias was determined based on the Luis Furuya-Kanamori (LFK) index and the Doi plot [21]. An LFK index within the range of outside ± 2, ± 2, and ± 1 is regarded as significantly/major asymmetrical, slightly/minor asymmetrical, and asymmetrical symmetrical (absence of publication bias), respectively. Furthermore, Cochrane’s Q test and inconsistency index (*I*^*2*^ statistics) was used to evaluate the magnitude of heterogeneity among included studies, considering *I*^2^ values of 0–25% as low, 25–50% as moderate and 50–75% as high heterogeneity [22]. A *p*-value less than 0.05 was defined as statistically significant. All procedures of statistical analyses were performed via meta and metasens packages in R version (3.6.1) [23].

## Results

### Characteristics of included studies

**Fig. 1:**
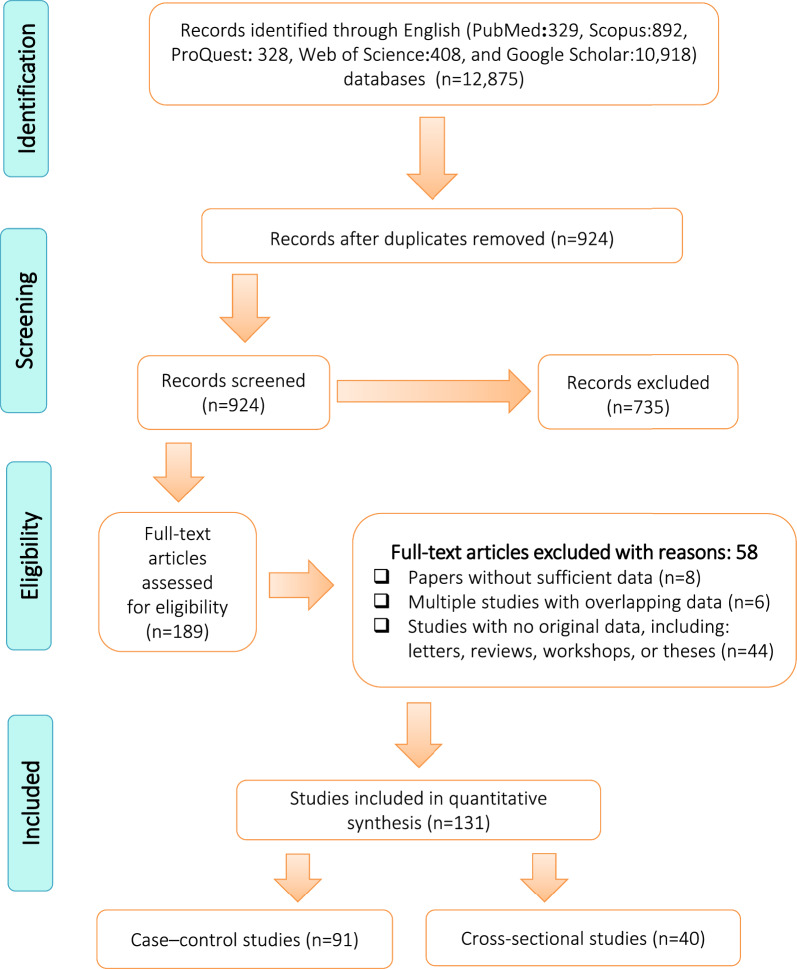
Flow diagram of the study design process

The initial database search yielded a total of 12,875 articles (Fig. 1). After screening and excluding duplicates, 131 articles (91 case–control and 40 cross-sectional studies) were found to be eligible and are included in this systematic review and meta-analysis. There were 91 case–control studies involving 18,626 cases and 18,312 controls (Tables 1 and 2), and 40 cross-sectional studies with 19,994 participants (Tables 4 and 5).

### The odds ratio/pooled prevalence

**Fig. 2:**
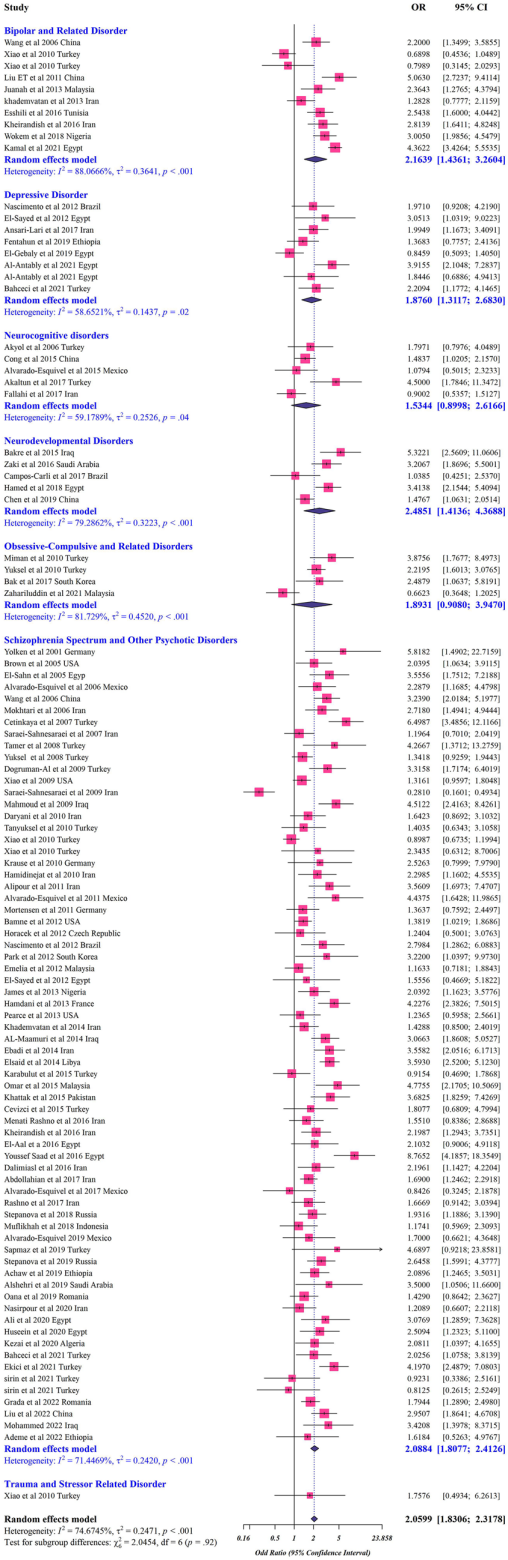
Forest plot of odds rations for relationship between prevalence of protozoan parasites and mental disorders in case–control studies

**Fig. 3:**
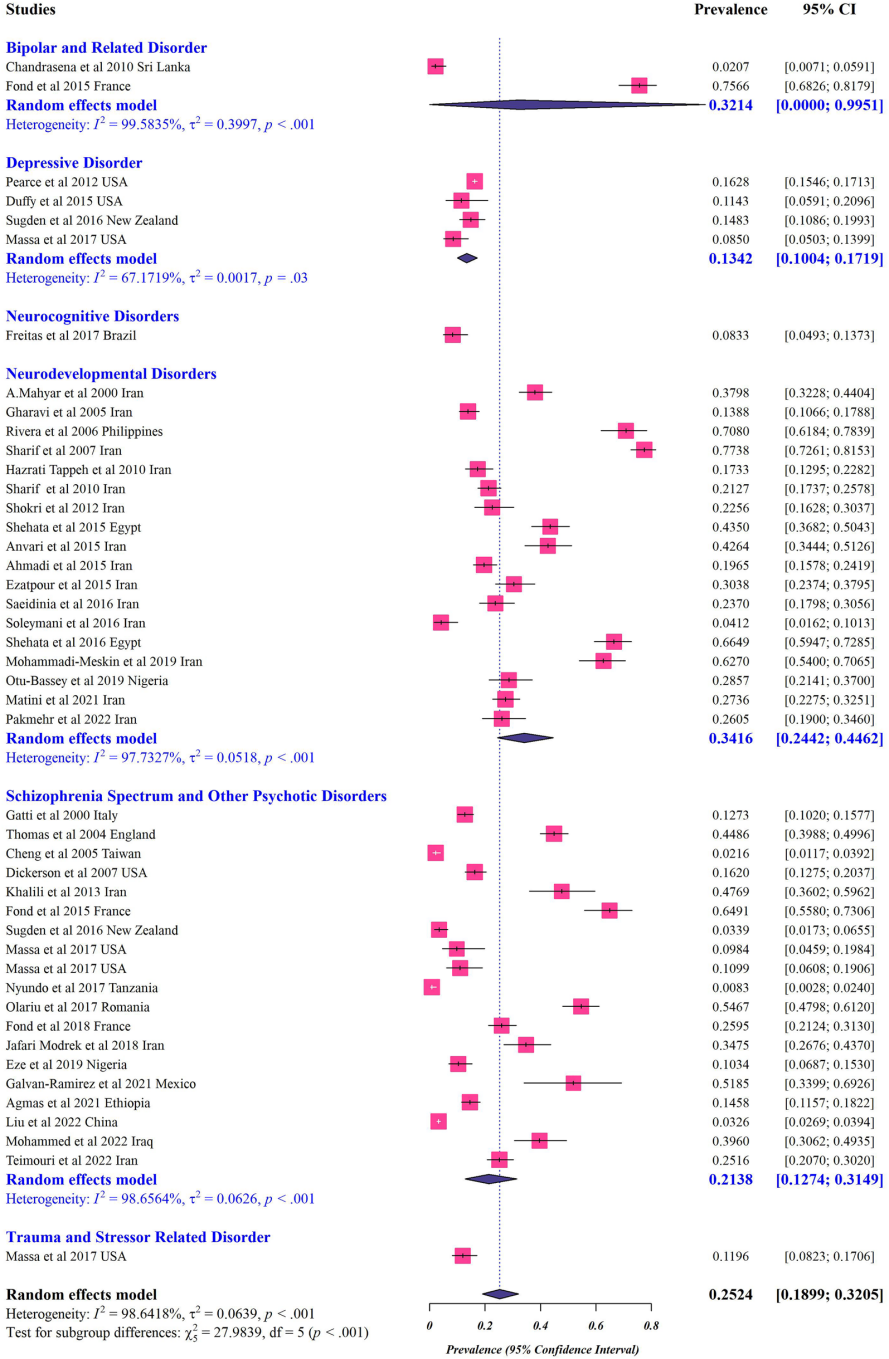
Forest plots for random-effects meta-analysis of the global prevalence of protozoan parasites among patients with mental health disorders based on cross-sectional studies (The boxes indicate the effect size of the studies (prevalence) and the whiskers indicate its confidence interval for corresponding effect size. There is no specific difference between white and black bars, only studies with a very narrow confidence interval are shown in white. In the case of diamonds, their size indicate the size of the effect, and their length indicate confidence intervals

The analysis based on case–control studies found a significant association between protozoan parasites and mental disorders (OR: 2.059, 95% CI 1.830–2.317) (Fig. 2, Table 2). The random-effects model for cross-sectional studies showed that the overall prevalence of protozoan parasites in patients with mental disorders was 0.252 (95% CI 0.189–0.320) (Fig. 3, Table 5). The heterogeneity was significant for both case–control (*I*^*2*^ = 74%; τ^2^ = 0.247; *p* < 0.001) and cross-sectional studies (*I*^*2*^ = 98%; τ^2^ = 0.063; *p* < 0.001) (Tables 2 and 5).

### Subgroup analysis

#### The odds ratio/pooled prevalence based on WHO regions

According to the WHO regions, our analyses of case–control studies revealed that the highest pooled OR was related to the Eastern Mediterranean Region (OR: 2.289, 95% CI 1.868–2.805) with heterogeneity (*I*^*2*^ = 79%; τ^2^ = 0.299; *p* < 0.001) (Table 2). The analyses of cross-sectional studies showed that protozoan parasitic infections were most prevalent in patients with mental disorders in the European Region (0.386, 95% CI 0.150–0.656) with heterogeneity (*I*^*2*^ = 98%; τ^2^ = 0.047; *p* < 0.001) (Table 5).

#### The odds ratio/pooled prevalence based on the type of the parasite

Subgroup analysis based on the type of protozoan parasite revealed the pooled OR of the higher risk of these parasites in patients with mental disorders in case–control studies (OR: 2.069, 95% CI 1.841–2.326) with heterogeneity (*I*^*2*^ = 73%; τ^2^ = 0.260; *p* < 0.001). However, the pooled OR of *Cyclospora cayetanensis* (4.719, 95% CI 1.352–16.474), followed by *Cryptosporidium parvum* (4.618, 95% CI 2.877–7.412) revealed a significantly higher risk of these parasites in patients with mental disorder compared to controls (Table 3).

The analysis based on cross-sectional studies showed that the pooled prevalence of different types of protozoan parasites was as follows: *T. gondii* (0.343, 95% CI 0.228–0.467) with heterogeneity (*I*^*2*^ = 99%; τ^2^ = 0.064; *p* < 0.001), *Cryptosporidium* spp. (0.087, 95% CI 0.005–0.242) with heterogeneity (*I*^*2*^ = 95%; τ^2^ = 0.032; *p* < 0.001), *Blastocystis hominis* (0.085, 95% CI 0.047–0.132) with heterogeneity (*I*^*2*^ = 93%; τ^2^ = 0.018; *p* < 0.001), *Entamoeba coli* (0.083, 95% CI 0.050–0.123) with heterogeneity (*I*^*2*^ = 92%; τ^2^ = 0.018; *p* < 0.001), *Cystoisospora belli* (0.076, 95% CI 0.022–0.156), *Cyclospora cayetanensis* (0.075, 95% CI 0.042–0.116), *E. histolytica / dispar* (0.064, 95% CI 0.011–0.151) with heterogeneity (*I*^*2*^ = 97%; τ^2^ = 0.067; *p* < 0.001), *Giardia lamblia* (0.062, 95% CI 0.040–0.088) with heterogeneity (*I*^*2*^ = 89%; τ^2^ = 0.008; *p* < 0.001), *Dientamoeba fragilis* (0.029, 95% CI 0.000–1.000) with heterogeneity (*I*^*2*^ = 95%; τ^2^ = 0.023; *p* < 0.001), *Endolimax nana* (0.029, 95% CI 0.006–0.067) with heterogeneity (*I*^*2*^ = 88%; τ^2^ = 0.017; *p* < 0.001), *Iodamoeba butschlii* (0.024, 95% CI 0.013–0.037) with heterogeneity (*I*^*2*^ = 53%; τ^2^ = 0.001; *p* < 0.001), *Chilomastix mesnili* (0.021, 95% CI 0.001–0.058) with heterogeneity (*I*^*2*^ = 77%; τ^2^ = 0.005; *p* < 0.001) (Table 6).

#### The odds ratio/pooled prevalence based on climatic variables

The estimates of pooled OR based on climatic variables showed that the highest rate was related to an annual precipitation range of 300–650 (OR: 2.297, 95% CI 1.700–3.103), humidity levels of 40–75% (OR: 2.194, 95% CI 1.917–2.510), annual rainfall of < 400 mm (OR: 2.256, 95% CI 1.844–2.761), and average temperatures of > 20 ℃ (OR: 2.281, 95% CI 1.850–2.811) (Table 2).

Moreover, our analyses of studies with cross-sectional design revealed that the highest pooled prevalence was observed for an annual precipitation of < 300 (0.270, 95% CI 0.192–0.355), humidity levels of < 40% (0.303, 95% CI 0.218–0.394), annual rainfall of < 400 mm (0.331, 95% CI 0.249–0.418), and the average temperatures of < 10 ℃ (0.448, 95% CI 0.398–0.499) (Table 5). The heterogeneity related to analyses based on each climatic variable in both study designs is presented in Tables 2 and 5.

#### The odds ratio/pooled prevalence based on diagnostic method

In terms of case–control studies, the highest rate of OR was related to studies that utilized a combination of EIA and Western blot methods (OR: 5.818, 95% CI 1.490–22.715) (Table 2).

Regarding cross-sectional studies, the highest pooled prevalence was associated with the combination of direct smear & PCR (0.708, 95% CI 0.618–0.783) (Table 5).

#### The odds ratio/pooled prevalence based on mean age and income level

The analyses of cross-sectional studies showed that protozoan parasites were most prevalent in patients with a mean age ranging from 15–20 years old (0.664, 95% CI 0.594–0.724) (Table 5).

According to our estimates regarding income level, the pooled OR (2.190, 95% CI 1.823–2.632) with heterogeneity (*I*^*2*^ = 77%; τ^2^ = 0.263; *p* < 0.001) and pooled prevalence (0.285, 95% CI 0.201–0.378) of protozoan parasitic infections was found to be highest in patients in lower-middle income regions with heterogeneity (*I*^*2*^ = 98%; τ^2^ = 0.060; *p* < 0.001) (Tables 2 and 5).

#### The odds ratio/pooled prevalence based on the type of the mental disorder

In terms of case–control studies, the association between different mental disorders and protozoan parasitic infections was as follows: neurodevelopmental disorders (OR: 2.485, 95% CI 1.413–4.368), bipolar and related disorders (OR: 2.163, 95% CI 1.436–3.260), schizophrenia spectrum and other psychotic disorders (OR: 2.088, 95% CI 1.807–2.412), obsessive–compulsive disorder (OCD) and related disorder (OR: 1.893, 95% CI 0.908–3.947), depressive disorder (OR: 1.876, 95% CI 1.311–2.683), neurocognitive disorders (OR: 1.534, 95% CI 0.899–2.616), and trauma and stressor related disorders (OR: 1.757, 95% CI 0.493–6.261) (Fig. 2).

Moreover, the analysis of cross-sectional studies revealed that the pooled prevalence based on the type of mental disorders was as follows: 0.341 (95% CI 0.244–0.446) in neurodevelopmental disorder with heterogeneity (*I*^*2*^ = 97%; τ^2^ = 0.051; *p* < 0.001), 0.321 (95% CI 0.000–0.995) in bipolar and related disorders with heterogeneity (*I*^*2*^ = 99%; τ^2^ = 0.399; *p* < 0.001), 0.213 (95% CI 0.127–0.314) in schizophrenia spectrum and other psychotic disorders with heterogeneity (*I*^*2*^ = 98%; τ^2^ = 0.062; *p* < 0.001), 0.134 (95% CI 0.100–0.171) in depressive disorder with heterogeneity (*I*^*2*^ = 67%; τ^2^ = 0.001; p < 0.001), 0.119 (95% CI 0.082–0.170) in trauma and stressor-related disorders with, and 0.083 (95% CI 0.049–0.137) in neurocognitive disorders (Fig. 3).

### Publication bias

**Fig. 4:**
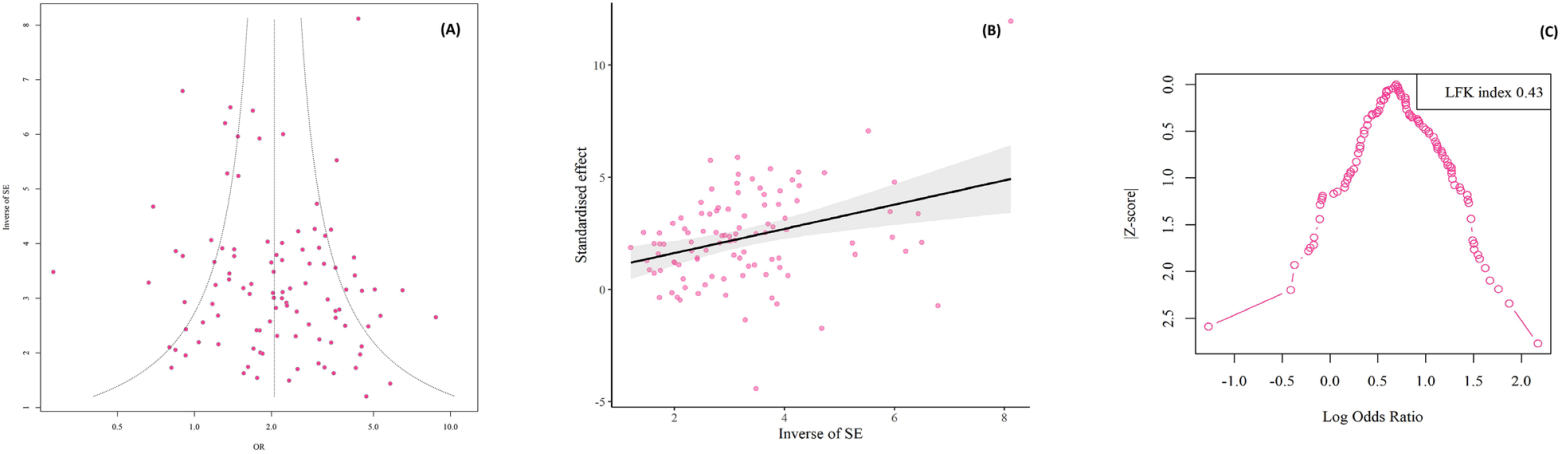
Egger's funnel plot and Begg's funnel plot to assess publication bias in studies evaluating of protozoan parasites among patients with mental health disorders based on case—control studies (Colored circles represent each study. The middle line is the effect size and the other two lines are the corresponding confidence ranges) (**A**, **B**). Doi plot for the global prevalence of intestinal protozoan parasites among patients with mental health disorders. A Luis Furuya -Kanamori (LFK) index 0.43 indicates no asymmetry (**C**)

**Fig. 5:**
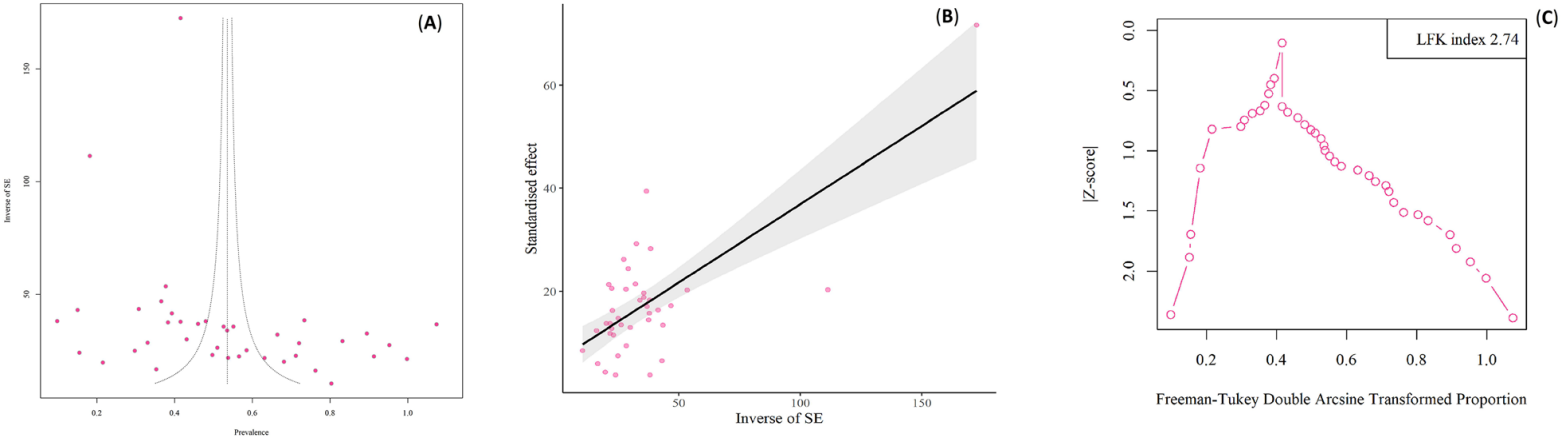
Egger's funnel plot and Begg's funnel plot to assess publication bias in studies evaluating of protozoan parasites among patients with mental health disorders based on cross-sectional studies (Colored circles represent each study. The middle line is the effect size and the other two lines are the corresponding confidence ranges) (**A**, **B**). Doi plot for the global prevalence of intestinal protozoan parasites among patients with mental health disorders. A Luis Furuya—Kanamori (LFK) index 2.74 indicates major asymmetry (**C**)

Substantial publication bias was detected in case–control studies, as indicated by Egger’s funnel plot (t = 1.03, p = 0.306) and Begg’s test (t = 1.02, p = 0.308). Additionally, the Doi plot test revealed no asymmetry (LFK index: 0.43) (Fig. 4). Substantial publication bias was detected in cross-sectional studies, as indicated by Egger’s funnel plot (t = 2.24, p = 0.025) and Begg’s test (t = 3.07, p = 0.004). Additionally, the Doi plot test revealed a major asymmetry (LFK index: 2.74) (Fig. 5).

### Meta-regression

**Fig. 6:**
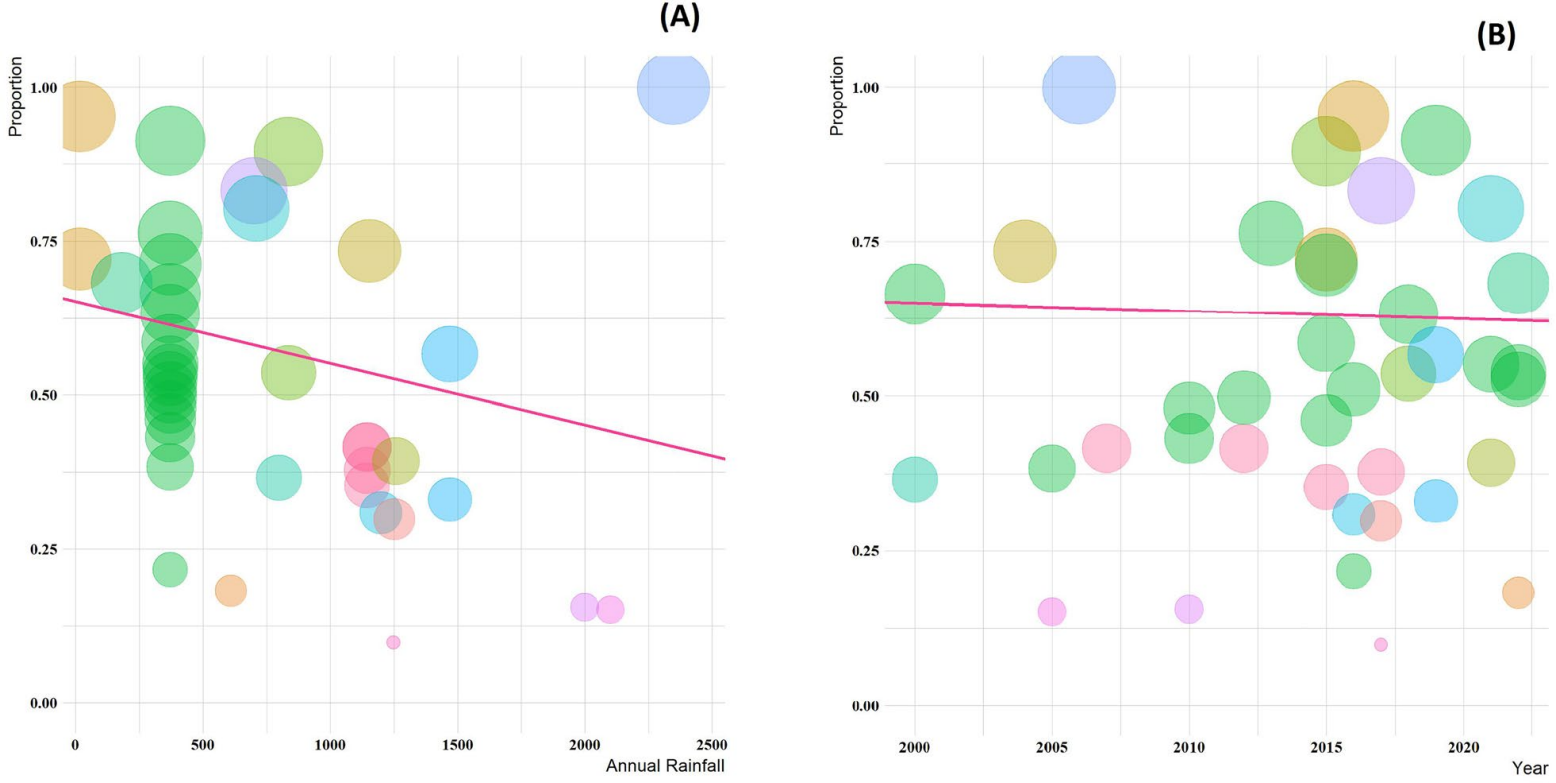
A meta-regression graph for the global prevalence of protozoan parasites among patients with mental health disorders based on annual rainfall (**A**), and year of publication (**B**) in cross-sectional studies. The pink line is the regression line, which was plotted based on the intercept and the slope of the regression model. The different color bubbles represent the countries under study and their sizes indicate the effect size of each study

**Fig. 7:**
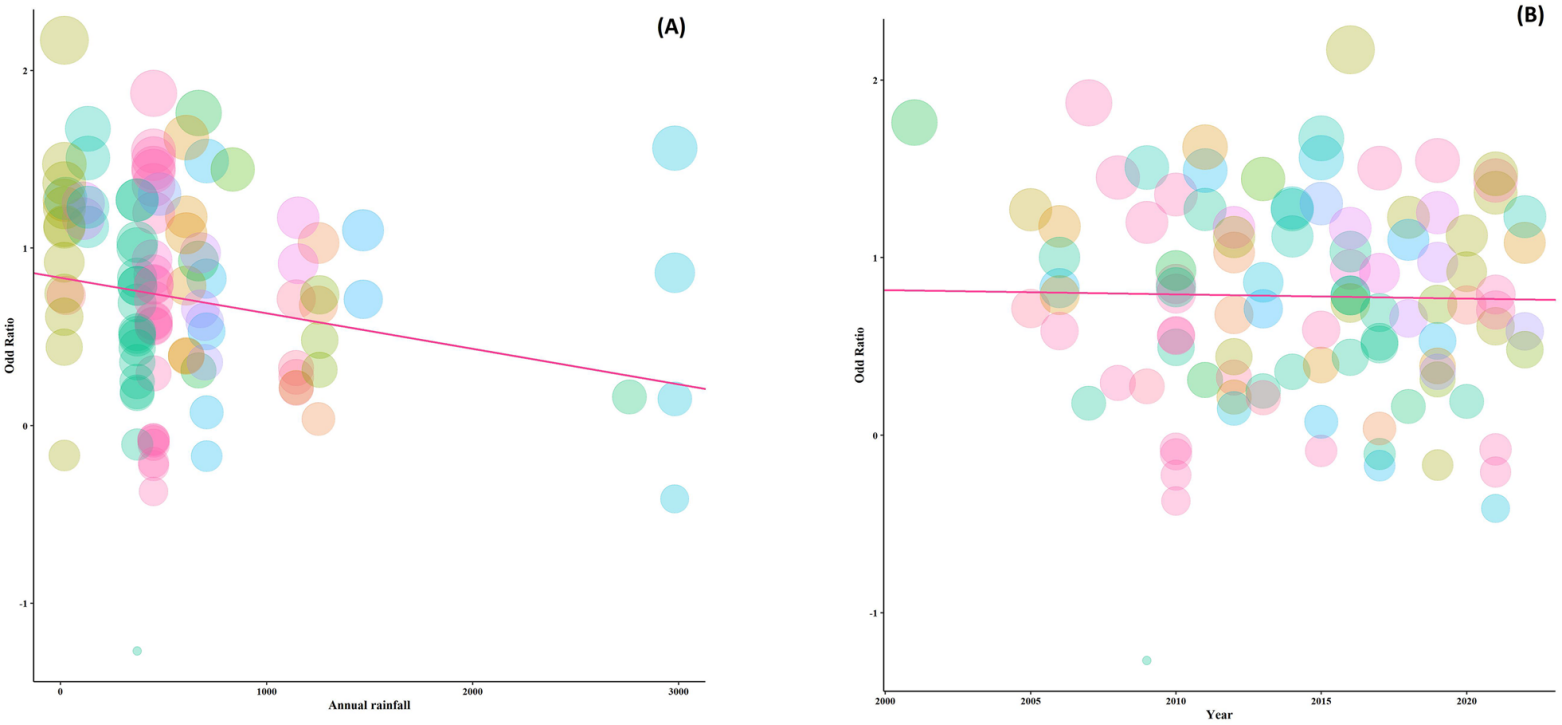
A meta-regression graph for the global prevalence of protozoan parasites among patients with mental health disorders based on annual rainfall (**A**), and year of publication (**B**) in case—control studies. The pink line is the regression line, which was plotted based on the intercept and the slope of the regression model. The different color bubbles represent the countries under study and their sizes indicate the effect size of each study

The results of the meta-regression analysis demonstrated that among all moderators, only annual rainfall, significantly affected the OR and the prevalence in studies with estimates of (slop = 0.6507, *p* < 0.0001) for a cross-sectional design and (slop = 0.8326, *p* < 0.0001) for a case–control studies (Figs. 6 and 7).

### QGIS3 map

**Fig. 8:**
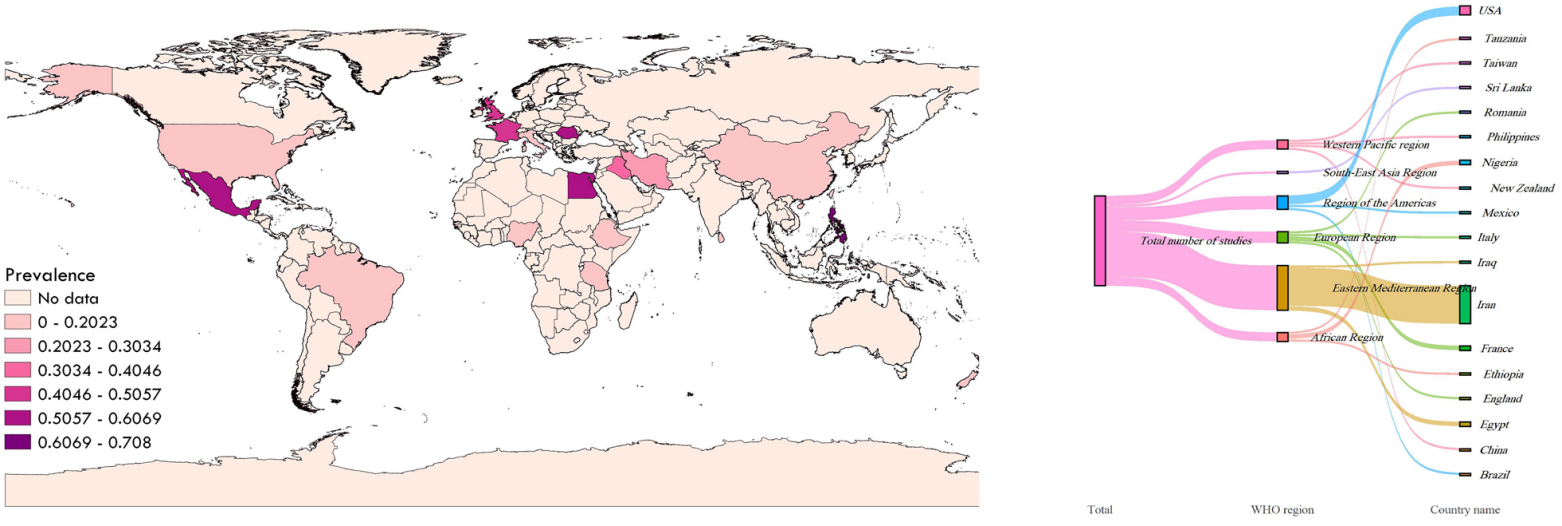
The prevalence of protozoan parasites among patients with mental health disorders based on cross-sectional studies in different geographical regions of the world

**Fig. 9:**
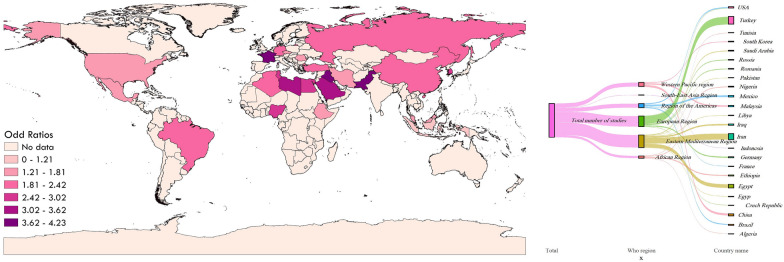
The prevalence of protozoan parasites among patients with mental health disorders based on case—control studies in different geographical regions of the world

To demonstrate the prevalence of protozoan parasites in patients with mental disorders in various geographical locations of the world, a map was created using QGIS3 software (https://qgis.org/en/site/) based on the included cross-sectional and case–control studies (Figs. 8 and 9).

## Discussion

The present systematic review and meta-analysis is the first to investigate an overlooked phenomenon concerning the status of protozoan parasitic infections and associated risk factors in patients with mental disorders through a comprehensive evaluation of the available data. The findings of our study revealed a pooled prevalence of 25.2% for protozoan parasites among the investigated patients. Notably, we also demonstrated that patients with mental disorders were about twofold more at risk of parasitic protozoan infections compared to healthy individuals.

Our analysis based on cross-sectional studies showed that, *T. gondii* was the most prevalent protozoan parasite among patients with mental health disorder.

In addition, the estimated pooled OR showed that that patients with mental disorder have a significantly higher risk of *C. cayetanensis* and *C. parvum* compared to control groups.

In the recent decades, a significant association has been identified between toxoplasmosis and a wide range of mental health diseases. Especially, this relationship has been well-documented in patients with bipolar disorder and schizophrenia. *T. gondii* is a widespread intracellular protozoan parasite with a neurotropic nature, which links it to mental and behavioral disorders. The chronic infection caused by the parasite is associated with formation of intracellular cysts in neurons and glial cells. Although, latent toxoplasmosis is commonly asymptomatic in immunocompetent patients, it is evidenced that it can trigger behavioral changes in mice and humans [24–27].

The experimental research demonstrated that *T. gondii* latent cysts distribute throughout the brain tissue in murine models. This experiment can raise the hypothesis that cysts formed by the parasite may affect frontal and limbic regions in humans resulting in emotional and behavioral changes [28]. Similarly, our subgroup meta-analysis of cross-sectional studies based on the type of mental disorder indicated that, patients with neurodevelopmental disorder followed by patients with bipolar and related disorders had the highest prevalence rate of toxoplasmosis. Previous studies showed that the seropositivity rates of anti-*Toxoplasma* antibodies were significantly higher in patients with neurodevelopmental disorders than in controls [29–32]. Furthermore, a meta-analysis study on 4021 patients diagnosed with bipolar disorder and 8669 healthy controls indicated that this disorder was associated with 1.34-fold higher risk of seropositivity for toxoplasmosis than healthy individuals [33]. As well, findings of a research conducted by Frye et al. [34], revealed that the inflammation which occur during toxoplasmosis infection may be one of the factors that have a role in bipolar disorder. Furthermore, it has been shown that a diminished long-term antibody response against *T. gondii* is associated with bipolar disorder and its subphenotypes, especially bipolar type I, non-early disease onset, and manic psychosis (OR: 1.33) [34].

In a recent analysis by Sutterland et al. [35], statistically significant ORs of the risk of anti-*T. gondii* IgG antibody have been reported in patients with bipolar disorder (OR: 1.52), and OCD patients (OR: 3.4). The investigation on relations between *T. gondii* and schizophrenia found a significant effect of seropositivity before onset and zero intensity. These findings revealed a potential link between toxoplasmosis and various psychiatric disorders, especially in schizophrenia which latent toxoplasmosis could be reactivated [35]. A comprehensive worldwide review discussed that *T. gondii* has the capacity to impact certain metabolic and developmental pathways, resulting in a modified susceptibility to the disease as a consequence [36]. Our estimates showed that patients with schizophrenia spectrum and other psychotic disorders were twofold at higher risk of protozoan parasite infections compared to the control group (OR: 2.08).

Nevertheless, the significance of intestinal parasites, particularly parasitic protozoa in patients with mental disorders, can not be underestimated. Our findings indicated the occurrence of intestinal parasitic infections, including both pathogenic and non-pathogenic types, among individuals diagnosed with mental disorders. Accordingly, our investigation found that *Cryptosporidium* spp., *C. cayetanensis*,* C. belli*, *E. histolytica/dispar*, and* G. lamblia* were among the pathogenic parasites in these patients. Several factors, including unhygienic lifestyle, common unhealthy behaviors (nail biting, improper food handling, and habits involving putting hands or objects into the mouth), unavailability of daily necessities and crowding (e.g., in mental health institutions/hospitals), pica, and mental disabilities, are considered to be contributing factors to this condition, which pose a greater risk of acquiring parasitic protozoan infections in patients with mental health disorders [37, 38].

Herein, we observed that parasitic protozoa were most prevalent in patients in lower-middle income regions with 2.1-fold higher risk of infection for mentally ill patients in these regions. Based on a review, mental illnesses are common in low and middle-income regions, with a pooled prevalence of 17.6% (15.5–20.0%) [14]. However, studies focused on the association of mental illnesses with parasitic diseases are limited in these countries.

Moreover, a geographical distribution in the prevalence of parasitic protozoa was observed in our meta-analysis. Accordingly, it has been shown that patients with mental disorders in the Eastern Mediterranean Region had a 2.28-fold higher risk of these parasites than healthy patients. In this region, there is a notable growth in population size, significant variations in socio-economic status between developed and developing nations, considerable migration trends, heightened demands for water resources, and a concerning degradation of ecosystems. The region is faced with the climate change issue, which elevates the likelihood of diseases transmitted through vectors, water, and food. Those facing the highest risk include individuals with lower socio-economic status, limited education, young children, the elderly, migrants, and those dealing with chronic health issues [39].

However, regarding cross-sectional studies, the highest pooled prevalence rate was detected in patients in European WHO region. This was similar to the finding of a global survey on the seroprevalence of toxoplasmosis in patients with mental and neurological disorders which found that the highest pooled prevalence of *T. gondii* IgG antibody was related to Europe (57%). Furthermore, this study showed that the overall global seroprevalence of *T. gondii* IgM antibody was higher in neuropsychiatric patients (6.78%) than in healthy controls (3.13%) [40].

In light of these reasons, comprehensive strategies that integrate both mental health and infectious disease management are imperative. These strategies should focus on raising awareness, improving healthcare access, addressing stigma, and promoting interdisciplinary collaboration between mental health professionals and infectious disease specialists. There is evidence regarding the significant relationship between climatic variables (particularly temperature, humidity, and rainfall) and the prevalence of intestinal protozoan diseases. The high temperatures and increased humidity in areas with humid and subtropical climates regarded as favorable conditions for the survival and transmission of protozoan parasites. Heavy rainfall can lead to water contamination and facilitate the spread of these parasites [5, 41]. Patients with mental disorders may be at higher risk of infection due to potential difficulties in maintaining personal hygiene and accessing healthcare, exacerbating their susceptibility. Understanding and addressing these relationships is crucial for the development of effective public health interventions and tailored care for this vulnerable group [12, 42].

## Limitations

This study faced some limitations that should be discussed. First, the number of studies for some subsets of mental disorders was limited, and for some of mental illnesses, only one article investigated the prevalence of protozoan parasites. Second, the heterogeneity of studies was high, and therefore, designing further studies with larger sample sizes and low heterogeneity is required to determine the contributing relationship between protozoan parasites and mental disorders.

## Conclusion

The present systematic review and meta-analysis have shed light on an overlooked connection between protozoan parasitic infections and mental disorders. Our study indicated that patients with mental disorders are at significantly higher risk of acquiring protozoan parasites than healthy individuals. We also demonstared that *T. gondii* as one of the most frequently observed parasite, has been extensively linked to a range of mental health conditions, with a strong correlation established in patients with bipolar disorder and schizophrenia.

This meta-analysis strengthens the growing body of evidence linking protozoan parasitic infections, particularly *T. gondii*, to mental disorders. The intricate interplay between infectious agents and mental health underscores the need for a multidisciplinary approach in understanding and managing these complex conditions. Recognizing and addressing these associations could have substantial implications for improving the overall well-being and mental health of affected individuals.

Considering the implications for public health arising from our findings, the current epidemiological data highlights the need for further research to explore the mechanisms underlying these connections and to develop effective strategies for prevention and intervention. Preventive interventions, regular screening programmes, and treatment for parasitic infections should be included in clinical care approaches applied to psychiatric patients, especially in specialized clinical services. It would be also beneficial if healthcare sectors in the psychiatry field consistently provided education on hygiene practices to improve the overall health of their patients.

### Supplementary Information

**Additional file 1: Table S1. **Quality assessment using the Newcastle–Ottawa scale modified for case-control studies.

**Additional file 2: Table S2.** Quality assessment using the Newcastle–Ottawa scale modified for cross-sectional studies.

## Data Availability

All data generated or analyzed during this study are included in this manuscript and Supplementary Files.

## Abbreviations

OR: Odds ratio
CI: Confidence interval
PRISMA: Preferred Reporting Items for Systematic Reviews and Meta-Analysis
LFK: Luis Furuya-Kanamori
*I*^*2*^: Inconsistency index
EIA: Enzyme Immunoassay
IFA: Immunofluorescence assay
OCD: Obsessive–compulsive disorder
