# Supplementary material for: A systematic review and meta-analysis of protozoan parasite infections among patients with mental health disorders: an overlooked phenomenon
Source: Gut Pathog. 2024 Jan 28;16:7. doi: 10.1186/s13099-024-00602-2 (PMC10822187; doi:10.1186/s13099-024-00602-2)
Supplement: Supplementary file 1 — Additional file 1: Table S1. Quality assessment using the Newcastle–Ottawa scale modified for case-control studies. [file 13099_2024_602_MOESM1_ESM.docx]

**Supplementary Table 1.**  Quality assessment using the Newcastle–Ottawa scale modified for case-control studies.

| No. | First author | Year | Selection  (maximum of 5 stars) | Comparability  (maximum of 2 stars) | Outcome  (maximum of 3 stars) | Total Score |
| --- | --- | --- | --- | --- | --- | --- |
| 1 | Yolken et al. | 2001 | *** | * | ** | 6 |
| 2 | Brown et al. | 2005 | *** | * | ** | 6 |
| 3 | El-Sahn et al | 2005 | *** | * | *** | 7 |
| 4 | Alvarado-Esquivel et al. | 2006 | ** | * | ** | 5 |
| 5 | Wang et al. | 2006 | *** | * | *** | 7 |
| 6 | Akyol et al. | 2006 | *** | * | ** | 6 |
| 7 | Mokhtari et al | 2006 | ** | * | ** | 5 |
| 8 | Cetinkaya et al. | 2007 | *** | * | *** | 7 |
| 9 | Saraei-Sahnesaraei et al. | 2007 | *** | * | ** | 6 |
| 10 | Tamer et al. | 2008 | ** | * | ** | 5 |
| 11 | Yuksel et al | 2008 | *** | ** | *** | 8 |
| 12 | Dogruman-Al et al. | 2009 | *** | * | ** | 6 |
| 13 | Xiao et al. | 2009 | ** | ** | *** | 7 |
| 14 | Saraei-Sahnesaraei et al | 2009 | *** | ** | ** | 7 |
| 15 | Mahmoud et al | 2009 | ** | * | ** | 5 |
| 16 | Daryani et al. | 2010 | *** | * | *** | 7 |
| 17 | Miman et al. | 2010 | ** | ** | *** | 7 |
| 18 | Tanyuksel et al. | 2010 | ** | * | ** | 5 |
| 19 | Xiao et al. | 2010 | **** | * | *** | 8 |
| 20 | Yuksel et al. | 2010 | *** | ** | *** | 8 |
| 21 | Krause et al. | 2010 | *** | * | ** | 6 |
| 22 | Hamidinejat et al | 2010 | *** | ** | *** | 8 |
| 23 | Alipour et al. | 2011 | *** | * | *** | 7 |
| 24 | Alvarado-Esquivel et al. | 2011 | ** | ** | *** | 7 |
| 25 | Liu ET et al. | 2011 | *** | ** | ** | 7 |
| 26 | Mortensen et al. | 2011 | ** | * | ** | 5 |
| 27 | Bamne et al. | 2012 | *** | * | *** | 7 |
| 28 | Horacek et al. | 2012 | *** | ** | *** | 8 |
| 29 | Nascimento et al. | 2012 | **** | ** | ** | 8 |
| 30 | Park et al. | 2012 | ** | * | ** | 5 |
| 31 | Emelia et al. | 2012 | *** | ** | *** | 8 |
| 32 | El-Sayed et al. | 2012 | *** | ** | *** | 8 |
| 33 | James et al. | 2013 | ** | ** | *** | 7 |
| 34 | Juanah et al. | 2013 | *** | ** | ** | 7 |
| 35 | khademvatan et al. | 2013 | **** | * | *** | 8 |
| 36 | Pearce et al. | 2013 | ** | * | ** | 5 |
| 37 | Hamdani et al. | 2013 | **** | ** | *** | 9 |
| 38 | Khademvatan et al. | 2014 | ** | * | ** | 5 |
| 39 | AL-Maamuri et al | 2014 | ** | * | ** | 5 |
| 40 | Ebadi et al | 2014 | *** | ** | *** | 8 |
| 41 | Elsaid et al | 2014 | ** | ** | *** | 7 |
| 42 | Karabulut et al. | 2015 | *** | * | ** | 6 |
| 43 | Omar et al. | 2015 | *** | * | ** | 6 |
| 44 | Cong et al. | 2015 | ** | * | ** | 5 |
| 45 | Khattak et al | 2015 | *** | * | *** | 7 |
| 46 | Cevizci et al | 2015 | **** | ** | *** | 9 |
| 47 | Bakre et al | 2015 | ** | * | ** | 5 |
| 48 | Alvarado-Esquivel et al | 2015 | *** | ** | *** | 8 |
| 49 | Esshili et al. | 2016 | *** | ** | *** | 8 |
| 50 | Kheirandish et al. | 2016 | ** | ** | *** | 7 |
| 51 | Zaki et al. | 2016 | **** | ** | *** | 9 |
| 52 | Menati Rashno et al. | 2016 | *** | ** | *** | 8 |
| 53 | El-Aal et a | 2016 | *** | ** | ** | 7 |
| 54 | Youssef Saad et al | 2016 | **** | ** | *** | 9 |
| 55 | Dalimiasl et al | 2016 | *** | * | *** | 7 |
| 56 | Abdollahian et al. | 2017 | *** | ** | *** | 8 |
| 57 | Alvarado-Esquivel et al. | 2017 | **** | ** | *** | 9 |
| 58 | Ansari-Lari et al. | 2017 | *** | ** | *** | 8 |
| 59 | Bak et al. | 2017 | *** | * | ** | 6 |
| 60 | Akaltun et al | 2017 | *** | ** | ** | 7 |
| 61 | Fallahi et al | 2017 | **** | * | *** | 8 |
| 62 | Rashno et al | 2017 | *** | * | *** | 7 |
| 63 | Campos-Carli et al | 2017 | **** | ** | *** | 9 |
| 64 | Hamed et al. | 2018 | ** | * | ** | 5 |
| 65 | Stepanova et al. | 2018 | *** | ** | ** | 7 |
| 66 | Muflikhah et al | 2018 | **** | ** | *** | 9 |
| 67 | Wokem et al | 2018 | *** | ** | *** | 8 |
| 68 | Alvarado-Esquivel | 2019 | *** | ** | ** | 7 |
| 69 | Chen et al. | 2019 | **** | * | *** | 8 |
| 70 | Fentahun et al. | 2019 | *** | * | ** | 6 |
| 71 | Sapmaz et al. | 2019 | *** | * | ** | 6 |
| 72 | Stepanova et al. | 2019 | ** | * | ** | 5 |
| 73 | Achaw et al | 2019 | **** | * | *** | 8 |
| 74 | Alshehri et al | 2019 | *** | ** | *** | 8 |
| 75 | Oana et al | 2019 | *** | * | ** | 6 |
| 76 | El-Gebaly et al | 2019 | *** | ** | *** | 8 |
| 77 | Nasirpour et al. | 2020 | *** | ** | ** | 7 |
| 78 | Ali et al | 2020 | *** | ** | ** | 7 |
| 79 | Huseein et al | 2020 | *** | ** | ** | 7 |
| 80 | Kezai et al | 2020 | ** | * | ** | 5 |
| 81 | Al-Antably et al. | 2021 | ** | * | ** | 5 |
| 82 | Bahceci et al. | 2021 | **** | ** | *** | 9 |
| 83 | Ekici et al | 2021 | *** | ** | ** | 7 |
| 84 | Kamal et al | 2021 | **** | * | *** | 8 |
| 85 | sirin et al | 2021 | *** | ** | *** | 8 |
| 86 | Zahariluddin et al | 2021 | ** | ** | *** | 7 |
| 87 | sirin et al | 2021 | *** | * | ** | 6 |
| 88 | Grada et al. | 2022 | ** | * | ** | 5 |
| 89 | Liu et al. | 2022 | **** | ** | *** | 9 |
| 90 | Mohammed | 2022 | *** | ** | ** | 7 |
| 91 | Ademe et al | 2022 | **** | * | *** | 8 |

*Indicates one criteria was followed, ** two criteria were followed, ***three criteria were followed, ****four criteria were followed, and ***** five criteria were followed
