# Supplementary material for: A systematic review and meta-analysis of protozoan parasite infections among patients with mental health disorders: an overlooked phenomenon
Source: Gut Pathog. 2024 Jan 28;16:7. doi: 10.1186/s13099-024-00602-2 (PMC10822187; doi:10.1186/s13099-024-00602-2)
Supplement: Supplementary file 2 — Additional file 2: Table S2. Quality assessment using the Newcastle–Ottawa scale modified for cross-sectional studies. [file 13099_2024_602_MOESM2_ESM.docx]

**Supplementary Table 2.** Quality assessment using the Newcastle–Ottawa scale modified for cross-sectional studies.

| No. | First author | Year | Selection  (maximum of 5 stars) | Comparability  (maximum of 2 stars) | Outcome  (maximum of 3 stars) | Total Score |
| --- | --- | --- | --- | --- | --- | --- |
| 1 | Gatti et al. | 2000 | **** | ** | ** | 8 |
| 2 | Mahyar et al. | 2000 | *** | * | ** | 6 |
| 3 | Thomas et al. | 2004 | *** | * | ** | 6 |
| 4 | Cheng et al. | 2005 | *** | * | *** | 7 |
| 5 | Gharavi et al. | 2005 | *** | ** | *** | 8 |
| 6 | Rivera et al | 2006 | **** | ** | ** | 8 |
| 7 | Dickerson et al. | 2007 | *** | * | *** | 7 |
| 8 | Sharif et al. | 2007 | *** | * | ** | 6 |
| 9 | Hazrati Tappeh et al. | 2010 | ** | * | ** | 5 |
| 10 | Sharif et al. | 2010 | *** | * | *** | 7 |
| 11 | Chandrasena et al | 2010 | ** | * | ** | 5 |
| 12 | Pearce et al. | 2012 | *** | ** | *** | 8 |
| 13 | Shokri et al. | 2012 | *** | * | ** | 6 |
| 14 | Khalili et al | 2013 | *** | ** | *** | 8 |
| 15 | Anvari et al. | 2015 | *** | * | *** | 7 |
| 16 | Duffy et al. | 2015 | **** | ** | ** | 8 |
| 17 | Ahmadi et al. | 2015 | *** | * | ** | 6 |
| 18 | Ezatpour et al. | 2015 | ** | * | ** | 5 |
| 19 | Fond et al. | 2015 | *** | * | *** | 7 |
| 20 | Shehata et al | 2015 | ** | * | ** | 5 |
| 21 | Saeidinia et al. | 2016 | *** | ** | ** | 7 |
| 22 | Soleymani et al. | 2016 | *** | * | ** | 6 |
| 23 | Sugden et al. | 2016 | *** | ** | *** | 8 |
| 24 | Shehata et al | 2016 | *** | * | *** | 7 |
| 25 | Freitas et al. | 2017 | ** | ** | *** | 7 |
| 26 | Massa et al. | 2017 | *** | ** | ** | 7 |
| 27 | Nyundo et al. | 2017 | **** | * | *** | 8 |
| 28 | Olariu et al | 2017 | *** | * | *** | 7 |
| 29 | Fond et al. | 2018 | ** | * | ** | 5 |
| 30 | Jafari Modrek et al | 2018 | ** | ** | *** | 7 |
| 31 | Mohammadi-Meskin et al. | 2019 | *** | ** | ** | 7 |
| 32 | Eze et al | 2019 | **** | * | *** | 8 |
| 33 | Otu-Bassey et al | 2019 | ** | ** | *** | 7 |
| 34 | Galvan-Ramirez | 2021 | *** | * | ** | 6 |
| 35 | Matini et al. | 2021 | *** | ** | ** | 7 |
| 36 | Agmas et al. | 2021 | *** | * | ** | 6 |
| 37 | Pakmehr et al. | 2022 | *** | ** | *** | 8 |
| 38 | Liu et al | 2022 | *** | * | ** | 6 |
| 39 | Mohammed et al | 2022 | *** | ** | *** | 8 |
| 40 | Teimouri et al | 2022 | *** | * | *** | 7 |

*Indicates one criteria was followed, ** two criteria were followed, ***three criteria were followed, ****four criteria were followed, and ***** five criteria were followed
